# mTOR hyperactivity mediates lysosomal dysfunction in Gaucher's disease iPSC-neuronal cells

**DOI:** 10.1242/dmm.038596

**Published:** 2019-10-01

**Authors:** Robert A. Brown, Antanina Voit, Manasa P. Srikanth, Julia A. Thayer, Tami J. Kingsbury, Marlene A. Jacobson, Marta M. Lipinski, Ricardo A. Feldman, Ola Awad

**Affiliations:** 1 Department of Microbiology and Immunology, University of Maryland School of Medicine, Baltimore, MD 21201, USA; 2 Department of Anesthesiology, University of Maryland School of Medicine, Baltimore, MD 21201, USA; 3 Department of Physiology, University of Maryland School of Medicine, Baltimore, MD 21201, USA; 4 University of Maryland Center for Stem Cell Biology and Regenerative Medicine, University of Maryland School of Medicine, Baltimore, MD 21201, USA; 5 Moulder Center for Drug Discovery Research, Temple University School of Pharmacy, Philadelphia, PA 19140, USA; 6 Department of Anatomy and Neurobiology, University of Maryland School of Medicine, Baltimore, MD 21201, USA

**Keywords:** Lysosomal storage disorder, *GBA1* mutations, TFEB, iPSC, mTORC1

## Abstract

Bi-allelic *GBA1* mutations cause Gaucher's disease (GD), the most common lysosomal storage disorder. Neuronopathic manifestations in GD include neurodegeneration, which can be severe and rapidly progressive. *GBA1* mutations are also the most frequent genetic risk factors for Parkinson's disease. Dysfunction of the autophagy-lysosomal pathway represents a key pathogenic event in *GBA1*-associated neurodegeneration. Using an induced pluripotent stem cell (iPSC) model of GD, we previously demonstrated that lysosomal alterations in GD neurons are linked to dysfunction of the transcription factor EB (TFEB). TFEB controls the coordinated expression of autophagy and lysosomal genes and is negatively regulated by the mammalian target of rapamycin complex 1 (mTORC1). To further investigate the mechanism of autophagy-lysosomal pathway dysfunction in neuronopathic GD, we examined mTORC1 kinase activity in GD iPSC neuronal progenitors and differentiated neurons. We found that mTORC1 is hyperactive in GD cells as evidenced by increased phosphorylation of its downstream protein substrates. We also found that pharmacological inhibition of glucosylceramide synthase enzyme reversed mTORC1 hyperactivation, suggesting that increased mTORC1 activity is mediated by the abnormal accumulation of glycosphingolipids in the mutant cells. Treatment with the mTOR inhibitor Torin1 upregulated lysosomal biogenesis and enhanced autophagic clearance in GD neurons, confirming that lysosomal dysfunction is mediated by mTOR hyperactivation. Further analysis demonstrated that increased TFEB phosphorylation by mTORC1 results in decreased TFEB stability in GD cells. Our study uncovers a new mechanism contributing to autophagy-lysosomal pathway dysfunction in GD, and identifies the mTOR complex as a potential therapeutic target for treatment of *GBA1*-associated neurodegeneration.

## Abstract (precis)

**Summary:** Using neuronopathic Gaucher's disease iPSCs, this study has identified the mTOR complex as a potential therapeutic target in *GBA1*-associated neurodegeneration.

## INTRODUCTION

Gaucher's disease (GD) is the most common lysosomal storage disorder (LSD). It is caused by bi-allelic mutations in the *GBA1* (also known as *GBA*) gene, which encodes the lysosomal enzyme β-glucocerebrosidase (GCase) (Jmoudiak and Futerman, 2005; Cox, 2010; Bendikov-Bar and Horowitz, 2012). GCase is responsible for the breakdown of the cellular glycolipids glucosylceramide (GluCer) and glucosylsphingosine (GluSph). Loss of GCase activity results in glycosphingolipid accumulation and cellular dysfunction (Brady et al., 1966; Xu et al., 2010). GD is characterized by various degrees of nervous system involvement depending on the severity of the particular *GBA1* mutation and other unknown factors (Mignot et al., 2013; Roshan Lal and Sidransky, 2017). Type 1 GD is characterized by absent or minimal neurological manifestations, whereas both types 2 and 3 GD develop neuropathological changes that can be fatal (Kaminsky et al., 1996; Bellettato and Scarpa, 2010). Type 2 (acute neuronopathic) GD patients develop severe rapidly progressive neurodegeneration leading to death during early childhood. Type 3 (chronic neuronopathic) GD patients also exhibit neurodegenerative changes, albeit with later onset and slower progression (Wong et al., 2004; Weiss et al., 2015; Orvisky et al., 2000). *GBA1* mutations are also the most frequent genetic risk factors for Parkinson's disease (Brockmann and Berg, 2014; Klein and Westenberger, 2012), and decreased GCase enzyme activity is linked to α-synuclein accumulation and Parkinson's disease pathogenesis (Almeida, 2012; Mazzulli et al., 2016; Schapira, 2015).

Dysfunction of the autophagy-lysosomal pathway represents a central pathogenic event in *GBA1*-associated neurodegeneration (Gan-Or et al., 2015; Zhang et al., 2015; Kinghorn et al., 2017). The autophagy-lysosomal pathway maintains cellular homeostasis by clearing protein aggregates and damaged organelles, which is critical for neuronal survival (Puyal et al., 2012; Kesidou et al., 2013; Menzies et al., 2017). Studies using neuronopathic GD mouse models and induced pluripotent stem cells (iPSCs) derived from GD patients indicated defective autophagic clearance and accumulation of undegraded proteins in neuronopathic GD neurons (Sun and Grabowski, 2010; Farfel-Becker et al., 2014; Awad et al., 2015; Liou et al., 2016; Schöndorf et al., 2014; Osellame et al., 2013). We have previously demonstrated that autophagy-lysosomal pathway alterations in neuronopathic GD iPSC neurons are linked to dysfunction of the transcription factor EB (TFEB), the master regulator of lysosomal biogenesis and autophagy. Both TFEB levels and stability were reduced in neuronopathic GD neurons, which resulted in lysosomal depletion and autophagy block (Awad et al., 2015). However, the mechanisms of *GBA1*-mediated TFEB dysfunction remain unknown.

TFEB controls the coordinated expression of lysosomal genes through binding to the coordinated lysosomal expression and regulation (CLEAR) element in their promoters (Settembre et al., 2012, 2011). Enhancing TFEB activity upregulates lysosomal functions and promotes protein clearance, which makes it a promising therapeutic target for neurodegenerative disorders (Kilpatrick et al., 2015; Decressac et al., 2013; Sardiello and Ballabio, 2009). TFEB is phosphorylated by multiple cellular kinases; the main one is the mammalian target of rapamycin (mTOR), which regulates TFEB subcellular localization and activity (Napolitano and Ballabio, 2016; Puertollano et al., 2018).

mTOR kinase is a key regulator of cellular growth and metabolism (Saxton and Sabatini, 2017a) downstream of the phophatidylinositol-3-kinase (PI3K)/AKT signaling network (Dibble and Cantley, 2015). mTOR exists in two functionally and structurally distinct complexes: mTORC1 and mTORC2 (Lipton and Sahin, 2014; Laplante and Sabatini, 2013). mTORC1 acts as a cellular nutrient sensor and is sensitive to growth factor stimulation, nutrients and cellular energy status (Sengupta et al., 2010). It conveys the nutrient-sensing signals to the lysosomes, thus coupling lysosomal functions to cellular energy demands (Dunlop and Tee, 2014; Rabanal-Ruiz and Korolchuk, 2018; Settembre and Ballabio, 2014a). To achieve this, mTORC1 phosphorylates TFEB on at least three serine residues, Ser122, Ser142 and Ser211, which in turn modulates TFEB subcellular localization and transcriptional activity (Puertollano et al., 2018; Napolitano and Ballabio, 2016). In a high nutrient/pro-growth state, mTORC1 is active and phosphorylates TFEB at Ser142 and/or Ser211, which prevents TFEB translocation from the cytoplasm to the nucleus through binding to the pan-14-3-3 scaffold protein (Martina et al., 2012). Upon mTORC1 inhibition due to cellular starvation or lysosomal stress, dephosphorylated TFEB is translocated to the nucleus where it upregulates its target genes (Settembre et al., 2012; Sardiello et al., 2009). It has been shown that mTORC1 also regulates TFEB stability and cellular abundance (Puertollano et al., 2018). TFEB phosphorylation at Ser142 and Ser211 targets inactive TFEB for proteasomal degradation through binding to the E3 ubiquitin ligase, STUB1 (Rao et al., 2017; Sha et al., 2017). Therefore, deregulation of mTORC1 kinase activity may alter TFEB phosphorylation status, which in turn affects TFEB levels and stability.

We have previously demonstrated altered TFEB-mediated lysosomal biogenesis and a block in macroautophagy (referred to hereafter as autophagy) in iPSC neurons derived from neuronopathic GD patients (Awad et al., 2015). In the present study, we investigated whether autophagy-lysosomal pathway dysfunction in neuronopathic GD iPSC neurons is mediated by deregulation of mTOR signaling. We found that mTOR is hyperactive in neuronopathic GD neuronal cells, as evidenced by increased levels of phospho-mTOR and excess phosphorylation of the mTORC1 downstream targets ribosomal protein S6 (RPS6) and the eukaryotic translation initiation factor 4E binding protein 1 (4EBP1; also known as EIF4EBP1). Treatment with GZ667161 (GZ-161), a glucosylceramide synthase inhibitor that prevents the synthesis of GluCer (Sardi et al., 2017), reduced p-RPS6 levels in neuronopathic GD cells, suggesting that mTORC1 hyperactivity was mediated by the abnormal accumulation of glycosphingolipids. We have previously demonstrated that treatment with the pharmacological mTORC1 inhibitor rapamycin resulted in autophagosome accumulation and decreased survival of neuronopathic GD neurons (Awad et al., 2015). In the present study, we found that treatment with Torin1, but not with rapamycin, upregulated lysosomal biogenesis in neuronopathic GD neurons. Torin1 also increased autophagosome-lysosome association and improved autophagic clearance in the mutant neurons. Moreover, we found that decreased TFEB stability in GD cells is mediated by mTORC1 hyperactivity. Our results suggest that autophagy-lysosomal pathway dysfunction in neuronopathic GD is linked to altered lipid-sensing by mTORC1, resulting in TFEB deregulation. This study also identifies the mTOR complex as a potential therapeutic target for treatment of *GBA1*-associated neurodegeneration.

## RESULTS

### Increased mTORC1 activity in neuronopathic GD iPSC neuronal progenitor cells (NPCs)

**Fig. 1.**
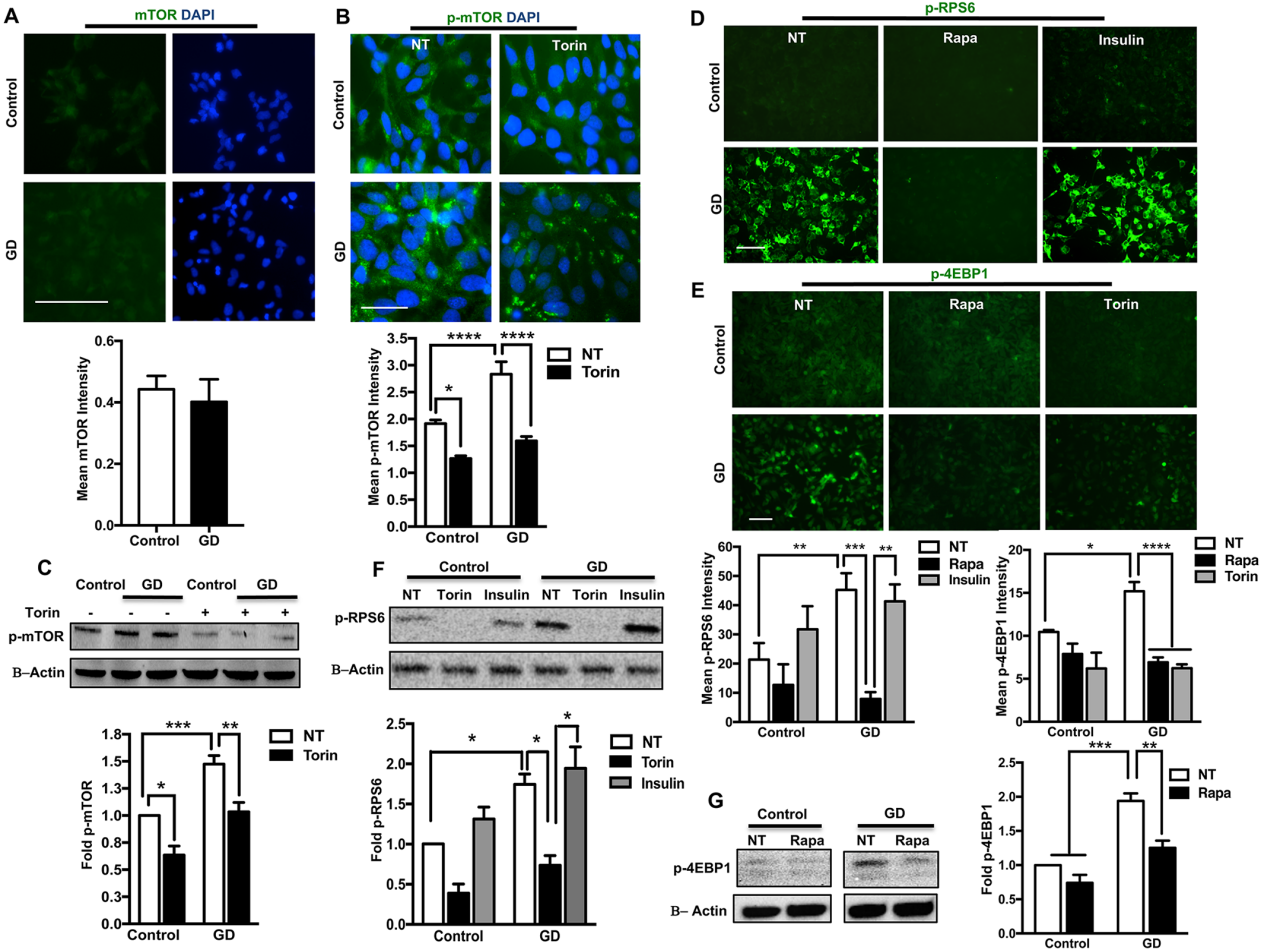
**Increased mTOR activity in GD** **iPSC NPCs.** (A) Representative immunofluorescence images of control and GD2a NPCs labeled for mTOR (green) or DAPI (blue). Bar graph shows mean mTOR fluorescence signal intensity in control and GD NPCs (GD2a, GD2b and GD3 combined). Data were collected from >100 cells per group, assayed in two different fields per experiment, in two independent experiments. *P*>0.05 between control and GD NPCs (Student's *t*-test). (B) Representative immunofluorescence images of control and GD2a NPCs labeled for phospho-mTOR-Ser2448 (p-mTOR) (green) and DAPI (blue). Cells were either untreated (NT) or treated with 100 nM Torin1 for 6 h. Bar graph shows mean p-mTOR fluorescence signal intensity in control and GD NPCs (GD2a and GD2b combined). Data were collected from >30 cells per group, assayed in three different fields in a representative experiment. (C) Representative western blot showing p-mTOR protein levels in control and GD NPCs derived from two GD iPSC lines (GD3 and GD2a). Cells were either untreated or treated with 100 nM Torin1 for 6 h. Also shown is β-actin loading control. Bar graph shows fold p-mTOR in treated relative to untreated control (GD2a, GD2b and GD3 NPCs combined), *n*=3-5 per group. (D) Representative immunofluorescence images of control and GD2a NPCs labeled for phospho-RPS6 Ser235/236 (p-RPS6). Cells were either untreated, treated with 200 nM rapamycin for 6 h or with 10 nM insulin for 30 min as indicated. (E) Representative immunofluorescence images of control and GD2b NPCs labeled for phospho-4EBP1 Thr37/46 (p-4EBP1). Cells were either untreated, treated with 200 nM rapamycin or 100 nM Torin for 6 h. Bar graph shows mean p-RPS6 and p-4EBP1 fluorescence signal intensity in control and GD NPCs (GD2a, GD2b and GD3 combined). Data were collected from >100 cells per group, assayed in two or three different fields in a representative experiment. (F) Representative western blot showing p-RPS6 protein levels in control and GD2a NPCs with or without Torin1 or insulin treatment. Also shown is β-actin loading control. Bar graph shows fold p-RPS6 (data from GD2a, GD2b and GD3 combined) relative to untreated control, *n*=3 per group. (G) Representative western blot showing p-4EBP1 levels in control and GD2a NPCs with or without rapamycin treatment. Also shown is β-actin loading control. Bar graph shows fold p-4EBP1 in GD NPCs (data from GD2a and GD2b combined) relative to untreated control, *n*=3 per group. Data are mean±s.e.m. *P<0.05, **P<0.005, ***P<0.0005, ****P<0.00005 (one-way ANOVA between indicated groups). Scale bars: 100 µm in A,D,E (magnification 20×); 50 µm in B (magnification 60×).

We have previously demonstrated lysosomal alterations in both NPCs and differentiated neurons derived from neuronopathic GD iPSC lines (Awad et al., 2015, 2017). To investigate whether these alterations are linked to mTOR deregulation, we examined mTOR levels and kinase activity in neuronopathic GD NPCs. NPCs were generated from control and neuronopathic GD iPSC lines as previously described (Awad et al., 2015). The neuronopathic GD iPSC lines were derived from two neuronopathic type 2 GD patients harboring the bi-allelic mutations L444P/Rec*Nci*I and W184R/D409H, and from one neuronopathic type 3 GD patient with L444P/L444P mutations. Immunofluorescence analysis showed no difference in total mTOR levels between control and mutant NPCs (Fig. 1A). However, levels of mTOR phosphorylated at Ser2448 (active p-mTOR) were markedly increased in mutant NPCs as indicated by increased fluorescence signal intensity (Fig. 1B). Treatment with the catalytic mTOR inhibitor Torin1 reduced p-mTOR fluorescence signal intensity in both control and mutant NPCs (Fig. 1B). Western blot analysis showed no difference in total mTOR levels between control and GD NPCs (Fig. S1A,B) but a significant increase in p-mTOR levels in neuronopathic GD NPCs, which were efficiently reduced by Torin1 treatment (Fig. 1C). As mTORC1 is involved in regulating lysosomal functions (Settembre et al., 2012; Rabanal-Ruiz et al., 2017), we compared mTORC1 activity in control and neuronopathic GD NPCs. We examined the levels of phosphorylated 4EBP1 (p-4EBP1) and phosphorylated RPS6 (p-RPS6). Both proteins are known downstream targets of mTORC1, and their phosphorylation status reflects mTORC1 kinase activity (Ikenoue et al., 2009). We found a marked increase in the fluorescence signal intensity of both p-RPS6 and p-4EBP1 in neuronopathic GD NPCs as compared with control cells (Fig. 1D,E). The levels of total RPS6 were not increased in neuronopathic GD NPCs as compared with control cells (Figs S1A,C and S2A). Treatment with the allosteric mTORC1 inhibitor rapamycin reduced the fluorescence signal intensity of both p-RPS6 and p-4EBP1 in mutant NPCs (Fig. 1D,E). Conversely, mTORC1 activation by insulin increased the levels of p-RPS6 (Fig. 1D,F). Western blot analysis showed significant increase in p-RPS6 and p-4EBP1 levels in neuronopathic GD NPCs (Fig. 1F,G). Torin1 treatment effectively reduced p-RPS6 and p-4EBP1 levels in both control and mutant NPCs (Fig. 1E,F; Fig. S2B); similarly, rapamycin significantly reduced mTORC1 activity in GD NPCs as shown by the decrease in p-4EBP1 levels following treatment (Fig. 1E,G). To confirm mTORC1 hyperactivity in neuronopathic GD NPCs, we used two additional control lines, the iPSC line (7TA) and the human embryonic stem cell (hESC) line (H9). Immunofluorescence analysis of NPCs generated from all three control lines showed similar baseline levels of p-mTOR, p-RPS6 and p-4EBP1 (Fig. S3A-D). Western blot analysis confirmed the comparable low levels of mTORC1 substrates in control NPCs compared with their increased levels in GD NPCs (Fig. S3E). Together, our data indicate that mTORC1 is hyperactive in neuronopathic GD NPCs, but is still responsive to pharmacological inhibition by either Torin1 or rapamycin.

### mTORC1 hyperactivity in neuronopathic GD NPCs is mediated by lipid substrate accumulation

**Fig. 2.**
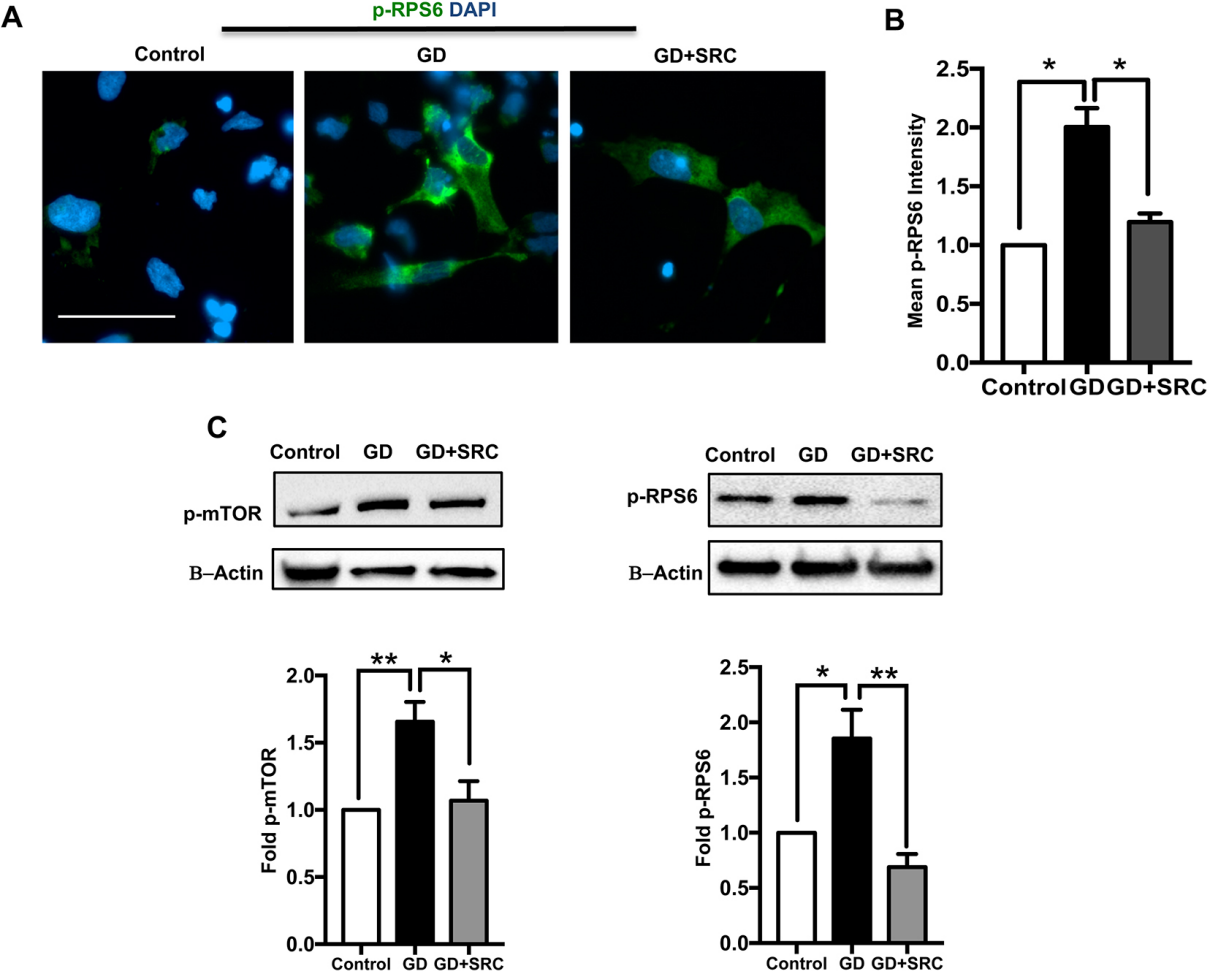
**Lipid substrate accumulation mediates mTOR hyperactivity in GD iPSC NPCs.** (A) Representative immunofluorescence images of control and GD2a NPCs co-labeled for p-RPS6 (green) and DAPI (blue). GD NPCs were either untreated or treated with 5 µM of the substrate reduction compound (SRC) GZ-161 for 72 h. (B) Quantitation of p-RPS6 fluorescence signal intensity in control and GD2a NPCs with and without GZ-161 treatment. Data from >100 cells per group, assayed in at least four different fields per experiment, in three experiments. Bar graph represents fold change in the mean p-RPS6 fluorescence intensity relative to control. (C) Representative western blot showing p-mTOR (left) and p-RPS6 (right) levels in control, GD2a and GD2a NPCs treated with the SRC. Also shown is β-actin loading control. Bar graphs represent fold p-mTOR and p-RPS6 in GD NPCs (data from GD2a and GD2b combined) relative to control, *n*=3-5 per group. Data are mean±s.e.m.*P<0.05 and **P<0.005 (one-way ANOVA between indicated groups). Scale bar: 50 µm (magnification 20×).

It is known that nutrient sufficiency promotes mTORC1 activation and phosphorylation of its downstream targets (Rabanal-Ruiz and Korolchuk, 2018). In GD, decreased GCase enzyme activity results in accumulation of its lipid substrate, GluCer, in mutant neurons (Schueler et al., 2003; Farfel-Becker et al., 2014). We have previously shown GluCer accumulation in GD iPSC neurons (Panicker et al., 2012). To test whether lipid substrate accumulation is responsible for the increased mTORC1 activity, we treated neuronopathic GD NPCs with the glucosylceramide synthase inhibitor GZ667161 (quinuclidin-3-yl-{2-[4′-fluor-(1,1′-biphenyl)-3-yl]propan-2-yl} carbamate) (GZ-161) for 72 h. This compound inhibits the glucosylceramide synthase enzyme, which catalyzes the synthesis of GluCer from ceramide, thus preventing its accumulation (Aerts et al., 2006; Lachmann and Platt, 2001). We then compared mTORC1 activity in treated and untreated NPCs. Our results indicated that GZ-161 treatment significantly reduced p-RPS6 levels in neuronopathic GD NPCs to almost control levels as shown by quantitation of p-RPS6 fluorescence signal intensity (Fig. 2A,B). Western blot analysis also showed that GZ-161 treatment of neuronopathic GD NPCs caused a significant reduction in both p-mTOR and p-RPS6 levels (Fig. 2C). Thus, our results suggest that increased mTORC1 activity in neuronopathic GD NPCs is mediated by the abnormal accumulation of glycosphingolipids in the mutant cells.

### Increased mTORC1 activity in neuronopathic GD iPSC neurons

**Fig. 3.**
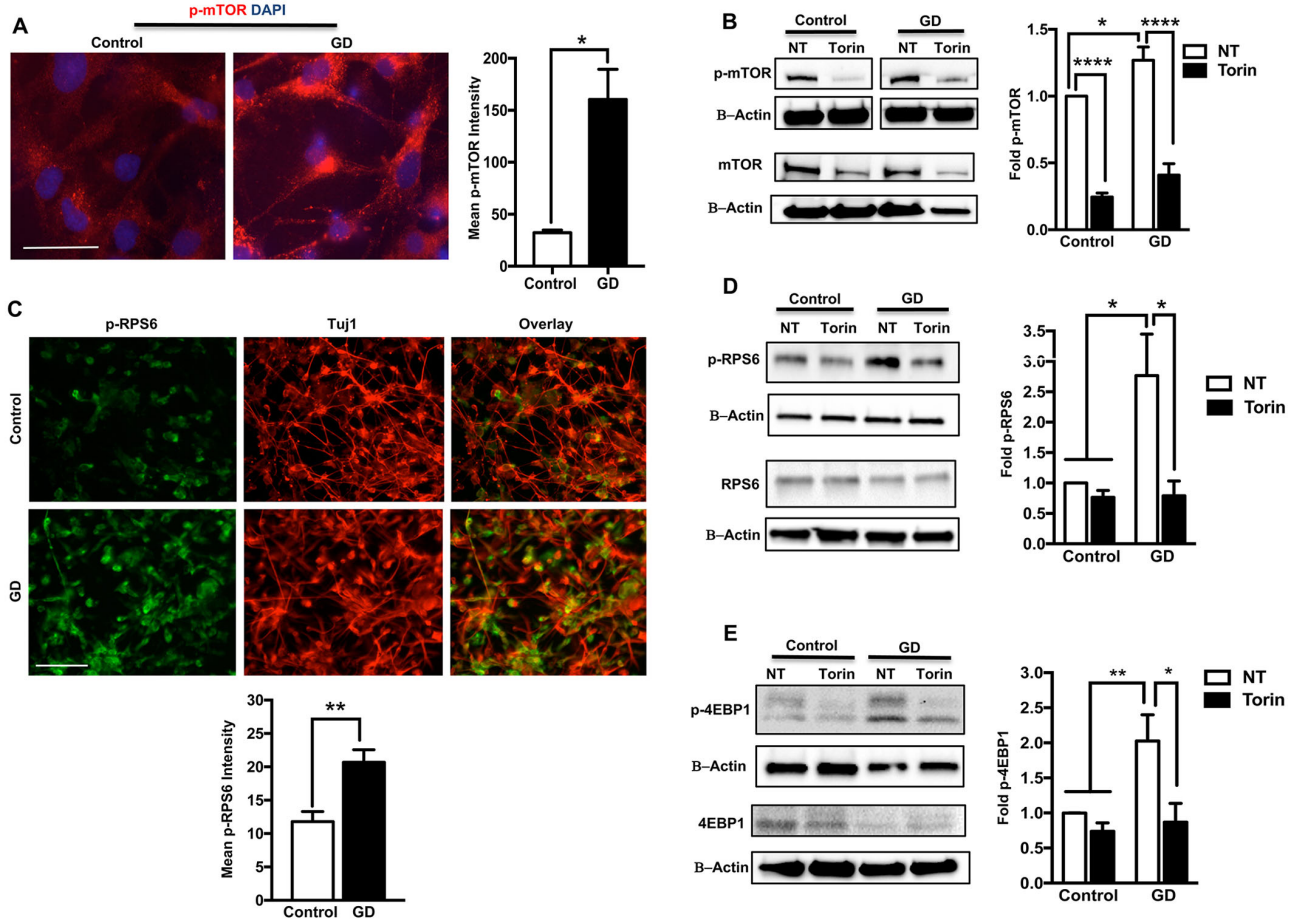
**Increased mTORC1 activity in GD iPSC neurons.** (A) Representative immunofluorescence images of control and GD3 neurons labeled for p-mTOR (red) and DAPI (blue). Bar graph (right) shows p-mTOR mean fluorescence signal intensity in control and GD neurons (GD2a and GD3 combined). Data were collected from >25 cells per group, assayed in two different fields, in a representative experiment. *P*<0.05 (Student's *t*-test). (B) Representative western blot showing p-mTOR (top) and mTOR (bottom) protein levels in control and GD2b neurons untreated (NT) or treated with 100 nM Torin1 for 6 h. Also shown is β-actin loading control. Bar graph represents fold p-mTOR in GD2 neurons (data from GD2a and GD2b combined) relative to untreated control, *n*=3-5 per group. (C) Representative immunofluorescence images of control and GD2b neurons labeled for p-RPS6 and Tuj1. The overlay of both markers is shown in the last panel. Bar graph (below) represents p-RPS6 mean fluorescence signal intensity in control and GD2 neurons (data from GD2a and GD2b combined). Data were collected from >100 cells per group, assayed in two to four different fields, in two experiments. *P*<0.005 (Student's *t*-test). (D) Representative western blot showing p-RPS6 (top) and RPS6 (bottom) levels in control and GD2a neurons with or without 100 nM Torin1 treatment for 6 h. Also shown is β-actin loading control. Bar graph represents fold p-RPS6 in GD2 neurons (data from GD2a and GD2b combined) relative to untreated control, *n*=3 per group. (E) Representative western blot showing p-4EBP1 (top) and 4EBP1 (bottom) protein levels in control and GD2a neurons with or without 100 nM Torin1 treatment for 6 h. Also shown is β-actin loading control. Bar graph represents fold p-4EBP1in GD2 neurons (data from GD2a and GD2b combined) relative to untreated control, *n*=3-4 per group. Data are mean±s.e.m. *P<0.05, **P<0.005, ****P<0.00005 (one-way ANOVA between indicated groups). Scale bars: 50 µm in A (magnification 60×); 100 µm in C (magnification 20×).

We then examined mTOR activity and p-mTOR levels in iPSC neurons differentiated from control and neuronopathic GD NPCs as previously described (Awad et al., 2015). We found that, similar to our findings in NPCs, levels of p-mTOR were significantly increased in neuronopathic GD neurons compared with control neurons (Fig. 3A,B). We also found that Torin1 treatment significantly reduced p-mTOR levels in both control and mutant neurons (Fig. 3B). We then examined mTORC1 activity by comparing the levels of p-RPS6 and p-4EBP1 in control and neuronopathic GD neurons. Both control and GD NPCs were differentiated to neurons as shown by the expression of the neuron-specific marker class III β-tubulin (Tuj1) (Fig. 3C). Immunofluorescence analysis showed increased fluorescence signal intensity of p-RPS6 in neuronopathic GD neurons compared with controls (Fig. 3C). Western blot analysis confirmed the significant increase in p-RPS6 but not total RPS6 levels in neuronopathic GD neurons (Fig. 3D). The level of p-4EBP1 was also significantly increased in neuronopathic GD neurons (Fig. 3E), and both p-RPS6 and p-4EBP1 levels were reduced to control levels in response to Torin1 treatment (Fig. 3D,E).

### Treatment of neuronopathic GD iPSC neurons with Torin1 upregulates lysosomal marker expression

**Fig. 4.**
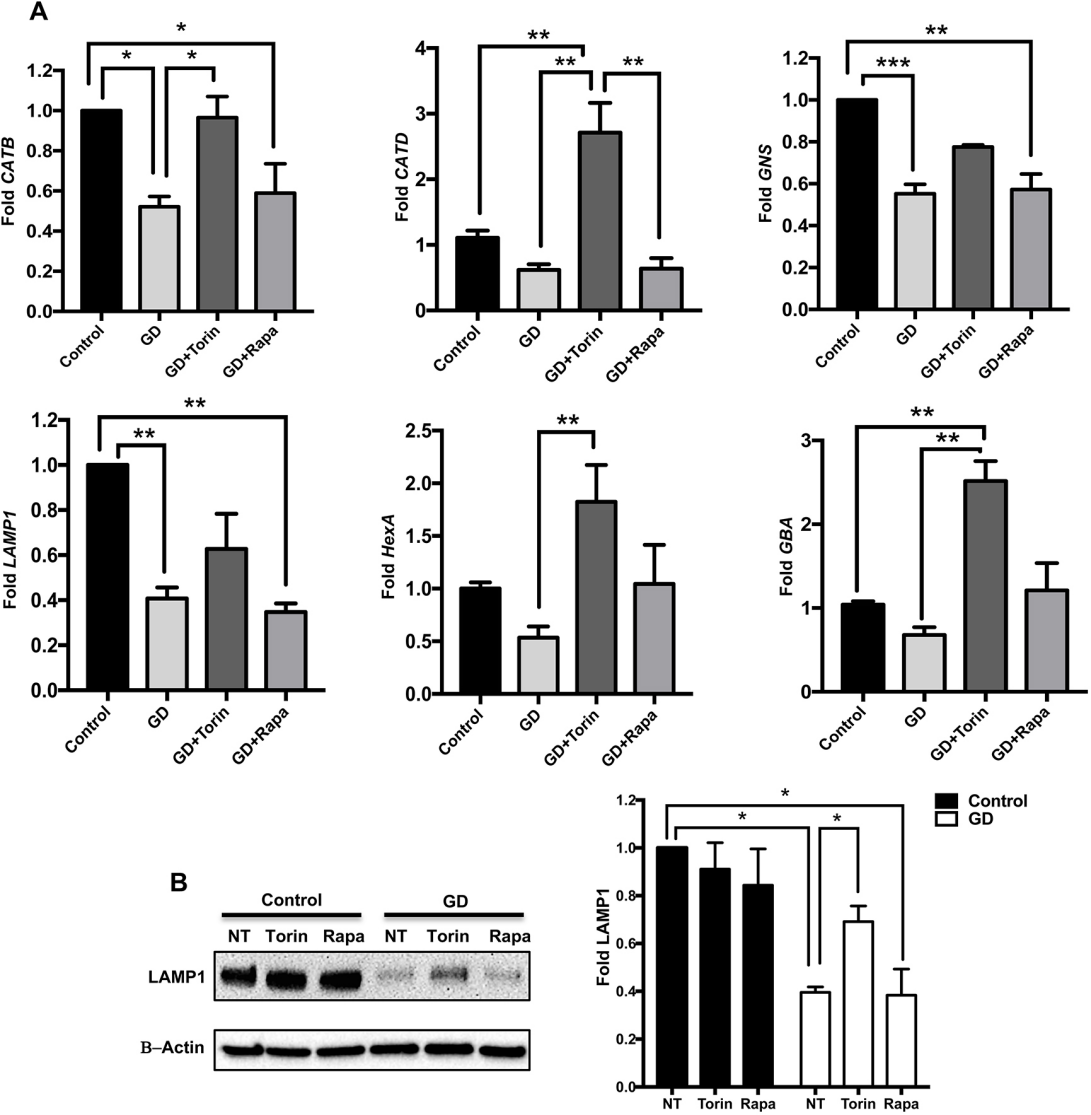
**Torin1 upregulates lysosomal marker expression in GD iPSC neurons.** (A) qRT-PCR analysis showing fold expression of lysosomal genes in control and GD2 neurons (data from GD2a and GD2b combined). GD neurons were untreated (NT) or treated with either 100 nMTorin1 or 200 nM rapamycin for 18 h. The lysosomal genes examined were cathepsin B (CATB), cathepsin D (CATD), *GNS*, *LAMP1*, *HEXA* and *GBA*, *n*=3-6 per group. (B) Representative western blot showing LAMP1 protein levels in control and GD2a neurons that were either untreated (NT) or treated with Torin1 or rapamycin. Also shown is β-actin loading control. Bar graph represents fold LAMP1 in control and GD2 neurons (data from GD2a and GD2b combined) relative to untreated control, *n*=3-4 per group. Data are mean±s.e.m. *P<0.05, **P<0.005, ***P<0.0005 (one-way ANOVA between indicated groups).

As dysfunction of the autophagy-lysosomal pathway is directly linked to neuronal loss in neuronopathic GD (Sun and Grabowski, 2010), it is important to elucidate its underlying mechanism. We have previously demonstrated altered TFEB-mediated lysosomal biogenesis and decreased lysosomal marker expression in neuronopathic GD iPSC neurons (Awad et al., 2015). To investigate whether those alterations are linked to mTOR hyperactivity, we examined the effects of pharmacological mTOR inhibition (using rapamycin or Torin1) on lysosomal gene expression in neuronopathic GD neurons by qRT-PCR analysis. The lysosomal genes examined were: *LAMP1*, *GBA1*, cathepsin D, cathepsin B, hexosaminidase A (*HEXA*) and glucosamine (N-acetyl)-6-sulfatase (*GNS*). These genes are known TFEB targets, which we have previously shown to be downregulated in neuronopathic GD iPSC neurons (Awad et al., 2015). We found that Torin1 significantly increased the expression levels of most of the lysosomal genes examined in treated versus untreated neuronopathic GD neurons (Fig. 4A), whereas rapamycin did not induce significant change in the expression levels of these genes (Fig. 4A), suggesting a differential effect of Torin1 and rapamycin on lysosomal biogenesis in neuronopathic GD neurons. To further explore this possibility, we compared the levels of the lysosomal-associated membrane protein 1 (LAMP1) in neurons treated with either Torin1 or rapamycin. As shown in Fig. 4B, western blot analysis indicated that LAMP1 levels were significantly reduced in mutant versus control neurons, and that Torin1, but not rapamycin, increased LAMP1 levels in neuronopathic GD neurons. Thus, our data suggest that the altered lysosomal biogenesis in neuronopathic GD neurons is linked to mTOR hyperactivity and that Torin1 is able to rescue this phenotype.

### Treatment with Torin1 induces TFEB-GFP nuclear translocation and upregulates lysosomal biogenesis in neuronopathic GD iPSC neurons

**Fig. 5.**
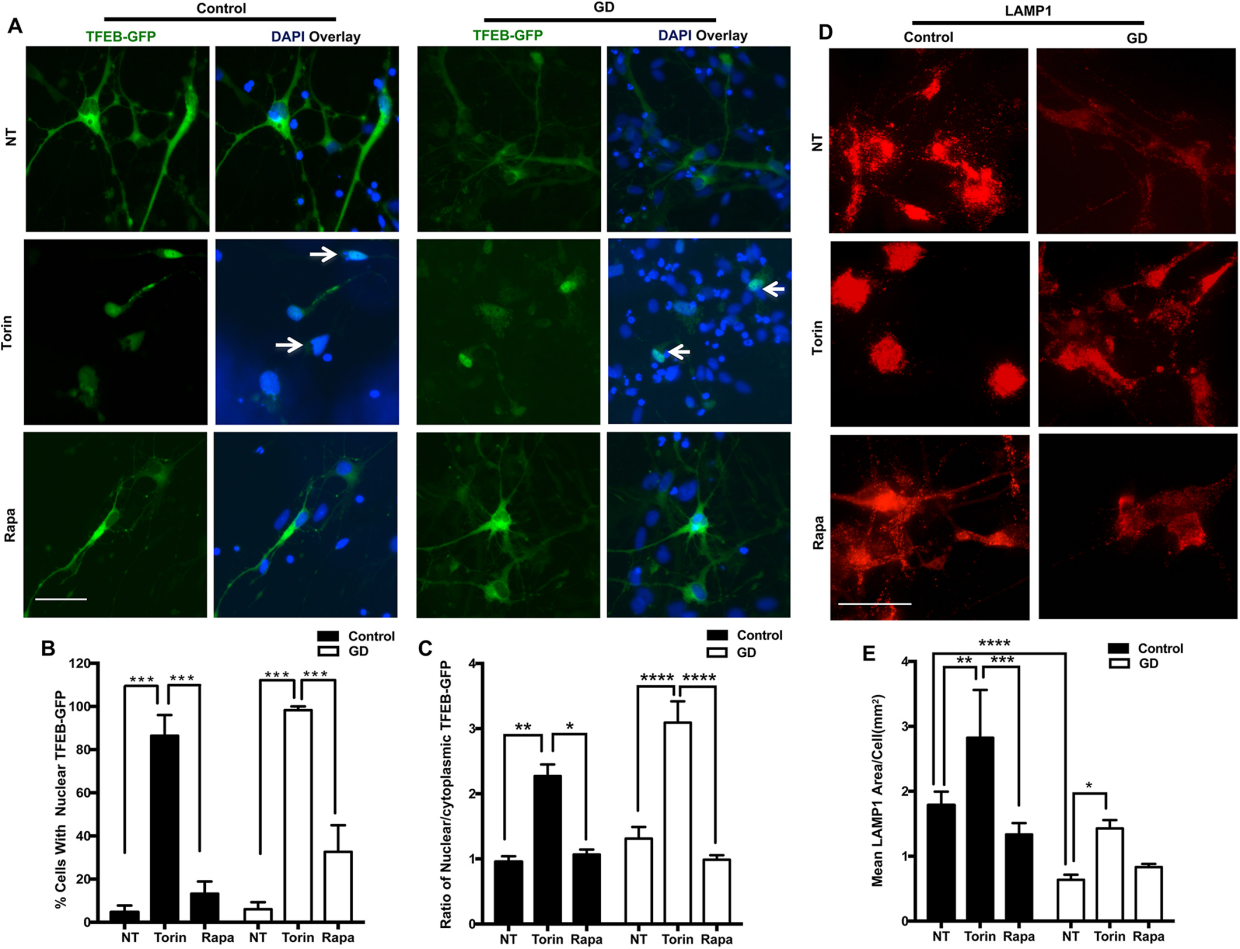
**Torin1 upregulates TFEB-mediated lysosomal biogenesis in GD iPSC neurons.** (A) Representative fluorescence images for control (left) and GD2a (right) neurons expressing TFEB-GFP fusion protein (green). Neurons were either untreated (NT) or treated with 100 nM Torin1 or 200 nM rapamycin for 18 h. Arrows point to the overlap of TFEB-GFP signal with nuclear DAPI (blue). (B) Quantitation of TFEB-GFP nuclear translocation in control and GD2 neurons (data from GD2a and GD2b combined) that were either untreated or treated with Torin1 or rapamycin. Bar graph represents the percentage of neurons with nuclear TFEB-GFP normalized to the number of GFP-expressing neurons in the same field, in three independent experiments. (C) Fluorescence quantitation of the ratio of nuclear/ cytoplasmic TFEB-GFP signal in control and GD2 neurons (data from GD2a and GD2b combined) that were either untreated or treated with Torin1 or rapamycin. Data from >50 cells, assayed in at least four different fields per group in two independent experiments. (D) Representative *z*-stack fluorescence images of control and GD2a neurons expressing TFEB-GFP fusion protein labeled for LAMP1. Neurons were either untreated or treated with 100 nM Torin1 or 200 nM rapamycin for 18 h. (E) Fluorescence quantitation of the LAMP1 area in control and GD2 neurons (data from GD2a and GD2b combined) that were either untreated or treated with Torin1 or rapamycin. Data from >50 cells, assayed in at least four different fields in three independent experiments. Data are mean±s.e.m. *P<0.05, **P<0.005, ***P<0.0005, ****P<0.00005 (one-way ANOVA between indicated groups). Scale bars: 50 μm in A (magnification 20×); 50 μm in D (magnification 60×).

To test whether the effect of mTOR inhibition on lysosomal biogenesis in neuronopathic GD neurons is mediated by TFEB, we overexpressed a TFEB-green fluorescent protein (GFP) fusion protein in control and neuronopathic GD neurons as previously described (Awad et al., 2015). We then treated neuronal cultures with either Torin1 or rapamycin and examined the changes in TFEB-GFP nuclear translocation, which is required for TFEB transcriptional activity. We found that Torin1 induced strong nuclear translocation of the TFEB-GFP fluorescence signal in both control and neuronopathic GD neurons compared with both untreated and rapamycin-treated cells (Fig. 5A). Almost all neurons treated with Torin1 had TFEB-GFP fluorescence signal primarily in the nucleus as shown by TFEB-GFP colocalized with nuclear DAPI staining (Fig. 5A,B). Quantitation of TFEB-GFP fluorescence intensity demonstrated an increase in the ratio of nuclear/cytoplasmic TFEB-GFP in control and neuronopathic GD neurons treated with Torin1, compared with both untreated and rapamycin-treated cells (Fig. 5C). Thus, consistent with previous reports, mTOR inhibition by Torin1 is more effective than rapamycin in inducing TFEB nuclear translocation (Settembre et al., 2012). We have previously shown that TFEB overexpression is unable to upregulate lysosomal biogenesis in neuronopathic GD neurons, except in the presence of recombinant GCase enzyme (Awad et al., 2015). Here, we wanted to test whether pharmacological inhibition of mTOR would overcome the requirement for active GCase and rescue TFEB-mediated lysosomal biogenesis in mutant neurons. To this end, we treated TFEB-GFP-expressing neurons with either Torin1 or rapamycin, and examined LAMP1 expression, which corresponds with lysosomal abundance. We noticed that Torin1, and to a lesser extent rapamycin, increased LAMP1 fluorescence intensity in neuronopathic GD neurons compared with untreated neurons (Fig. 5D). Quantitation of LAMP1 signal demonstrated that Torin1 treatment significantly increased LAMP1 area in both control and neuronopathic GD neurons (Fig. 5E). Thus, our data indicate that mTOR inhibition with Torin1 is able to enhance TFEB-mediated lysosomal biogenesis in neuronopathic GD neurons.

### Treatment with Torin1 improves autophagic clearance in GD iPSC neurons

**Fig. 6.**
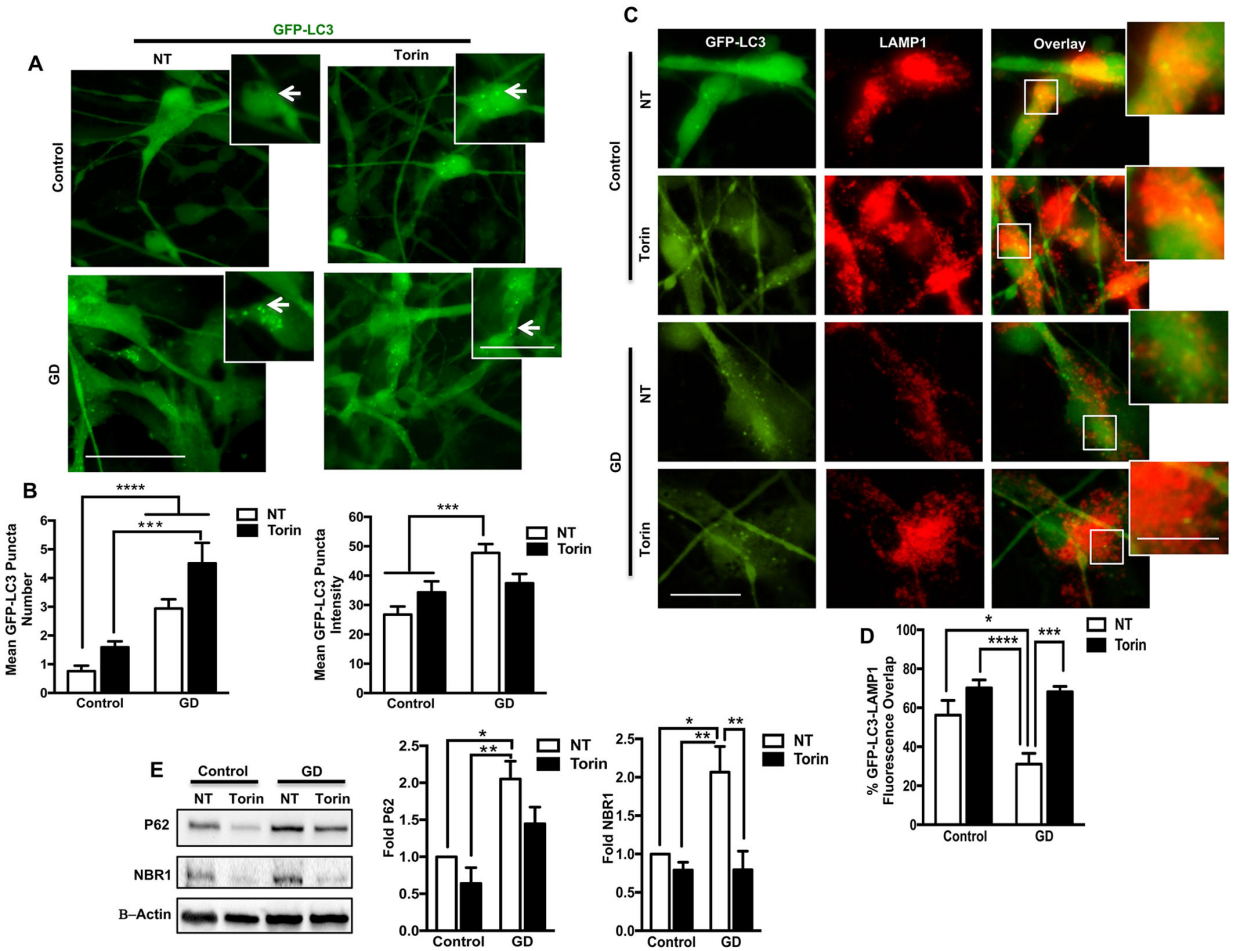
**Torin1 improves autophagic clearance in GD iPSC neurons.** (A) Representative fluorescence images for control and GD2b neurons, expressing GFP-LC3 fusion protein. Neurons were either untreated (NT) or treated with 100 nM Torin1 for 18 h. Insets are enlargement of a small area in each panel. Arrows point to GFP-LC3-labeled puncta. (B) Fluorescence quantitation of GFP-LC3 puncta in GD2 neurons (data from GD2a and GD2b combined) with and without Torin1 treatment. The bar graphs show average GFP-LC3 puncta number (left) and average GFP-LC3 puncta fluorescence intensity (right). (C) Representative fluorescence images for control and GD2a neurons, expressing GFP-LC3 fusion protein (green) and LAMP1 (red). Neurons were either untreated or treated with 100 nM Torin1 for 18 h. The merged image of GFP-LC3 puncti and LAMP1-labeled lysosomes is also shown. Insets to the right are enlargement of the boxed areas to show the fluorescence signal colocalization. (D) Quantitation of the percentage colocalization of GFP-LC3 puncti and LAMP1 fluorescence signal in control and GD2 neurons (data from GD2a and GD2b combined) with and without Torin1 treatment for 18 h. Data from >100 cells per group assayed in at least four different fields in two independent experiments. (E) Representative western blot showing p62 and NBR1 protein levels in control and GD2b neurons that were either untreated or treated with 100 µm Torin1 for 18 h. Also shown is β-actin loading control. Bar graphs show fold p62 (left) and NBR1 (right) in GD neurons (Data from GD2a, GD2b and GD3 combined) relative to the untreated control, *n*=3-4 per group. Data are mean±s.e.m. *P<0.05, **P<0.005, ***P<0.0005, ****P<0.00005 (one-way ANOVA between indicated groups). Scale bars: 50 µm in A (magnification 40×); 25 µm in A (inset); 25 µm in C (magnification 20×); 10 µm in C (inset).

Next, we tested whether the observed upregulation of TFEB-mediated lysosomal biogenesis by Torin1 would also improve autophagic clearance in neuronopathic GD neurons. We expressed a GFP-LC3 fusion protein in control and neuronopathic GD neurons as previously described (Awad et al., 2015) and treated the cells with Torin1. We then examined the number and fluorescence intensity of GFP-LC3 puncta, which corresponds with autophagosome abundance (Klionsky et al., 2012). There was a significant increase in both the number and fluorescence intensity of GFP-LC3 puncta in neuronopathic GD compared with control neurons (Fig. 6A,B), indicating autophagosome accumulation in the basal state. We have previously demonstrated that this autophagosome accumulation in neuronopathic GD neurons is due to defects in lysosomal degradation rather than an increase in autophagy flux (Awad et al., 2015). Treatment with Torin1 did not cause a statistically significant increase in either the number or the fluorescence intensity of GFP-LC3 puncta in control or mutant neurons compared with the corresponding untreated cells (Fig. 6B). This suggests that autophagy induction by Torin1 did not result in excessive autophagosome accumulation in the mutant neurons, which is opposite to the effect of rapamycin that we have previously reported (Awad et al., 2015). Similar results were obtained when we compared endogenous levels of LC3II (which corresponds to autophagosome abundance) in NPCs by western blot analysis (Fig. S4A). We then examined the effect of Torin1 on autophagosome association with the lysosomes. We labeled GFP-LC3-expressing neurons with anti-LAMP1 antibody and determined the percentage of GFP-LC3 puncta colocalization with LAMP1 using fluorescence signal quantitation (Fig. 6C; Fig. S5). As shown in Fig. 6D, we found a significant increase in the fraction of GFP-LC3 puncta colocalized with LAMP1 in neuronopathic GD neurons treated with Torin1 compared with untreated cells, indicating increased autophagosome-lysosome association in response to treatment. To test whether the enhanced autophagosome-lysosomal association induced by Torin1 would improve autophagic clearance, we examined levels of the autophagic substrate p62/SQSTM1 (p62) protein in neuronopathic GD neurons expressing the GFP-LC3 fusion protein. Immunofluorescence analysis showed an increase in p62 fluorescence signal intensity in neuronopathic GD neurons compared with controls, which was significantly reduced by treatment with Torin1 (Fig. S4B,C). To further explore the effect of Torin1 on autophagic clearance in neuronopathic GD cells; we measured endogenous protein levels of p62 and NBR1 (another autophagic substrate that also targets ubiquitinated proteins for autophagic degradation). Western blot analysis showed a significant increase in the basal levels of p62 and NBR1 proteins in neuronopathic GD neurons, indicative of an autophagic defect, and that treatment with Torin1 reduced the levels of both proteins (Fig. 6E). Both p62 and NBR1 proteins are known to promote autophagic degradation of the ubiquitinated targets (Kirkin et al., 2009), thus a decrease in their levels reflects efficient degradation via the lysosomes. Taken together, our results indicate that treatment with Torin1 improves both lysosomal biogenesis and autophagic clearance in neuronopathic GD neurons.

### Decreased TFEB stability in neuronopathic GD NPCs is linked to mTOR hyperactivity

We have previously shown decreased TFEB protein levels in neuronopathic GD iPSC-derived neurons and NPCs (Awad et al., 2015, 2017). To further confirm the effect of decreased GCase activity on TFEB levels, we treated a human neuroglioma cell line (H4), which stably expresses a TFEB-GFP fusion protein with the pharmacological irreversible GCase inhibitor conduritol B-epoxide (CBE). This model enables us to directly monitor the effect of decreasing GCase activity by treatment with CBE on TFEB levels. We then examined GFP fluorescence signal intensity, which corresponds with TFEB levels. We found that as early as 48 h after CBE treatment, TFEB-GFP fluorescence signal intensity significantly declined in treated versus untreated H4 cells (Fig. S6A). Treatment with the proteasome inhibitor (PSI) Clasto-lactacystin β-lactone restored the GFP signal intensity in CBE treated H4 cells, suggesting that the decline in TFEB level was due to excess TFEB degradation by the proteasome (Fig. S6B). We also found a significant increase in p-mTOR levels in GFP-TFEB-expressing H4 cells in response to CBE treatment (Fig. S6C), suggesting that decreased GCase activity affects mTOR phosphorylation status.

**Fig. 7.**
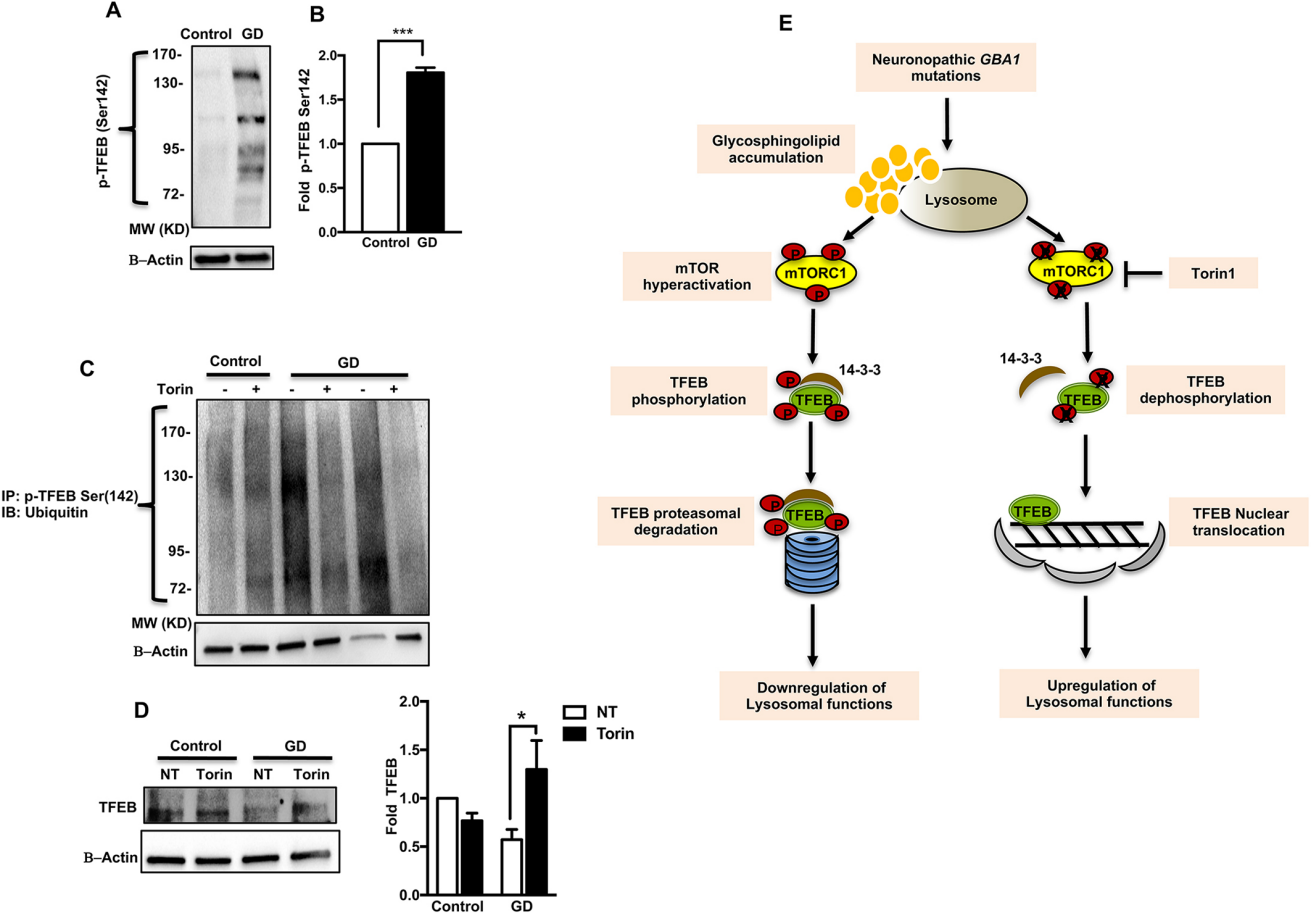
**Increased p-TEFB(Ser142) level in GD iPSC NPCs.** (A) Representative western blot showing p-TEFB(Ser142) levels in control and GD2a NPCs that were treated with the proteasome inhibitor Clasto-lactacystin β-lactone for 18 h. Also shown is β-actin loading control. (B) Bar graph shows western blot quantitation of fold p-TEFB(Ser142) level in GD2 NPCS (data from GD2a and GD2b combined) relative to control, *n*=3 per group. (C) Representative immunoprecipitation for control, GD2a, and GD2b NPCs using p-TFEB(Ser142) antibody and probed with anti-ubiquitin antibody. Cells were treated with the proteasome inhibitor for 18 h and either untreated or co-treated with Torin1 for 8 h. β-Actin loading levels in the input lysates are also shown. (D) Representative western blot (left) showing total TEFB level in control and GD2a NPCs. Cells were treated with the proteasome inhibitor for 18 h and were either untreated or co-treated with Torin1 for 8 h. Bar graph (right) shows western blot quantitation of total TEFB in GD NPCs (GD2a and GD3) relative to untreated control, *n*=3 per group. (E) Schematic for a proposed mechanism of TFEB dysfunction in neuronopathic GD, in which glycosphingolipid accumulation leads to increased mTORC1 activity. mTORC1 hyperactivation results in increased TFEB phosphorylation, which targets TFEB for proteasomal degradation. mTOR inhibition by Torin1 stabilizes TFEB and allows its nuclear translocation, thus upregulating lysosomal functions. Data are mean±s.e.m. *P<0.05, ***P<0.0005 (Student's *t*-test).

It is known that TFEB phosphorylation by mTORC1 at Ser142 targets inactive TFEB for proteasomal degradation (Sha et al., 2017). We wanted to examine whether the decreased TFEB stability in neuronopathic GD cells is linked to mTOR hyperactivity. First, we confirmed the decrease in TFEB levels in GD NPCs by western blot analysis (Fig. S6D). Then we treated NPCs with a PSI to prevent proteasomal degradation of phosphorylated TFEB and compared the levels of p-TFEB(Ser142) in control and neuronopathic GD NPCs using a phospho-specific antibody. Western blot analysis of protein lysates from neuronopathic GD NPCs treated with a PSI showed multiple p-TFEB(Ser142) bands of high molecular weight (Fig. 7A). Western blot quantification showed a significant increase in p-TFEB (Ser142) levels in neuronopathic GD NPCS compared with control cells (Fig. 7B). We then examined whether polyubiquitination of p-TFEB is responsible for this ladder pattern detected in neuronopathic GD NPCs lysates. To this end, we treated control and mutant NPCs with a PSI and performed immunoprecipitation of p-TFEB(Ser142) followed by western blot analysis using anti-Ubiquitin antibody. Our results showed increased ubiquitination of p-TFEB in neuronopathic GD NPCs compared with control cells (Fig. 7C). The intensity of the polyubiquitinated bands in neuronopathic GD cells was efficiently reduced by Torin1 treatment, suggesting that mTOR inhibition in neuronopathic GD NPCs decreased p-TFEB ubiquitination (Fig. 7C). Moreover, western blot analysis of total TFEB levels in NPCs that were treated with PSI and were either untreated or co-treated with Torin1 showed a significant increase in total TFEB levels in GD NPCs treated with Torin1 compared with untreated cells (Fig. 7D). This further supports that Torin1 increases TFEB stability in GD cells. Taken together, our results support a model in which neuronopathic *GBA1* mutations result in lipid substrate accumulation and increased mTORC1 activity. mTORC1 hyperactivity causes excess TFEB phosphorylation, which in turn targets TFEB for ubiquitination and proteasomal degradation. This model is shown in Fig. 7E.

## DISCUSSION

In this study we provide evidence that autophagy-lysosomal pathway dysfunction in neuronopathic GD iPSC-derived neurons is, at least in part, mediated by increased mTOR activity. mTOR is a highly conserved serine-threonine kinase that exists in two multiprotein complexes, mTORC1 and mTORC2 (Laplante and Sabatini, 2009). mTORC1 integrates multiple signals such as growth factors, energy status and nutrient abundance, to control cellular growth and metabolism (Saxton and Sabatini, 2017b; Sengupta et al., 2010). It is now known that mTORC1 also regulates the anabolic-catabolic equilibrium of many classes of lipids (Ricoult and Manning, 2013; Menon et al., 2017). This is achieved, in part, by controlling autophagic degradation of cellular lipids, or lipophagy (Antikainen et al., 2017; Settembre and Ballabio, 2014b). This process involves coordination between lysosomal nutrient-sensing machinery by mTORC1 and TFEB activity (Settembre and Ballabio, 2014b; Jaishy and Abel, 2016). Thus, it is likely that the abnormal lipid storage in LSDs disrupts the signaling regulation of this machinery. In support of this idea, studies have demonstrated mTOR signal dysregulation in various LSD models such as Fabry disease, Pompe disease and mucopolysaccharidoses (Ward et al., 2016; Bartolomeo et al., 2017; Lim et al., 2017). In this study, we found that treatment of neuronopathic GD NPCs with the substrate reduction compound GZ-161 normalized mTORC1 activity, suggesting that the increase in mTORC1 activity was indeed mediated by the abnormal glycosphingolipid accumulation. Similarly, it has been shown that GlcCer synthase inhibitors act on Akt-mTOR signaling and reduce phosphorylation of RPS6 (Natoli et al., 2010). Substrate reduction treatment also increased lysosomal abundance and improved autophagic flux in mouse primary neurons in an mTOR-dependent manner (Shen et al., 2014). In addition, GZ-161 has been shown to cross the blood brain barrier in neuronopathic GD mouse models (Cabrera-Salazar et al., 2012), and preventing GlcCer accumulation provides neuroprotective effects in some models of LSD (Stein et al., 2012; Ashe et al., 2011). Substrate reduction compounds are shown to provide clinical improvement in type 1 GD patients (Mistry et al., 2017; Zimran et al., 2018). Our study suggests that it may also be an effective approach to restore autophagy-lysosomal pathway functions in neuronopathic GD.

It is known that the recruitment and retention of mTORC1 to the lysosome is required for its activation (Yao et al., 2017). Thus, the defective lysosomes in LSDs may diminish rather than increase mTORC1 activity. However, it has been shown that in some LSD models, lysosomal storage enhances rather than inhibits mTORC1 signaling (Bartolomeo et al., 2017; Vidal-Donet et al., 2013). The co-existence of mTORC1 hyperactivation and lysosomal dysfunction has also been found in some genetic models of Parkinson's disease, which was linked to impaired TFEB activity (Bento et al., 2016). Our study suggests that in neuronopathic GD, mTORC1 hyperactivation may also occur without fully functional lysosomes. The potential involvement of mTOR signaling deregulation in neuronopathic GD was also reported in mouse models. Dasgupta et al. have previously demonstrated increased expression of mTOR pathway mRNAs in the brains of neuronopathic GD mice (Dasgupta et al., 2015). However, our findings contradict previous reports using other genetic models of *GBA1* deficiency, which show mTORC1 signaling attenuation, not activation. Magalhaes et al. reported that in primary neurons from *GBA1* transgenic mice and in fibroblasts from Parkinson's disease patients with *GBA1* mutations, there was altered lysosomal recycling and a decrease in phosphorylated S6K levels (Magalhaes et al., 2016). Similarly, in a *Drosophila* model of neuronopathic GD, mTOR signaling was found to be downregulated in the brains of mutant flies. Yet, rapamycin treatment partially rescued some of the observed phenotypes, raising the possibility that inhibiting the activity of mTOR may be of therapeutic value (Kinghorn et al., 2016). As these reports on the relation between mTOR activity and GCase enzyme were carried out in different models of *GBA1* deficiency, the reasons behind this apparent discrepancy are unclear. One possibility is differential species-dependent (mouse and fly versus human) and cell type-dependent (fibroblasts versus NPCs and neurons) regulation of the mTOR and TFEB activity in response to GlcCer accumulation. Further studies are needed to clarify the role of mTORC1 in *GBA1*-associated neurodegeneration, and to explore the different therapeutic approaches for treatment of those conditions.

We have previously demonstrated altered TFEB-mediated lysosomal biogenesis in neuronopathic GD neurons (Awad et al., 2015). mTORC1 is a key upstream negative regulator of TFEB (Settembre et al., 2012), thus our data indicate that TFEB alterations in iPSC neuronopathic GD neurons are likely mediated by mTORC1 hyperactivity. A similar correlation between mTOR hyperactivity and TFEB downregulation was also reported in AMPK-deficient embryoid bodies, which exhibit elevated mTOR signaling (Young et al., 2016). Young et al. found that AMPK^−/−^ embryoid bodies have reduced levels of TFEB and alterations in TFEB phosphorylation status. They also found that treatment with a pharmacological mTOR inhibitor reversed TFEB hyperphosphorylation status in those knockout embryoid bodies (Young et al., 2016). It is known that when mTORC1 is active, it phosphorylates TFEB on Ser142 and/or Ser211, which prevents TFEB nuclear translocation and sequesters it in the cytosol (Puertollano et al., 2018; Napolitano and Ballabio, 2016). However, although mTORC1 was hyperactive in our GD iPSC neurons, we did not detect excess cytoplasmic sequestration of TFEB. Instead, total TFEB levels were reduced and, when detected, TFEB was mainly in the nucleus (Awad et al., 2015). These findings might be explained by studies showing that mTORC1 also regulates TFEB stability (Puertollano et al., 2018). mTORC1 phosphorylation of Ser142 and Ser211 residues targets inactive phosphorylated TFEB for ubiquitination and proteasomal degradation (Sha et al., 2017). In agreement with this report, we found increased levels of phosphorylated TFEB Ser142 in neuronopathic GD NPCs, which can explain the decreased TFEB levels and stability that we previously reported (Awad et al., 2015).

Our results showed that treatment of neuronopathic GD neurons with the pharmacological mTOR inhibitor Torin1 rescued TFEB-mediated lysosomal biogenesis and improved autophagic clearance. This is in contrast to our previous results demonstrating that rapamycin is unable to rescue TFEB function in neuronopathic GD neurons, instead resulting in increased cell death. It is known that rapamycin is an allosteric mTOR inhibitor and only suppresses part of mTORC1 function, whereas Torin1 is a catalytic inhibitor that completely suppresses mTOR activity (Thoreen and Sabatini, 2009; Thoreen et al., 2009). As Torin1 inhibits both mTORC1 and mTORC2 activity (Zheng and Jiang, 2015), we cannot rule out the possibility of mTORC2 involvement in our results. However, it is known that many mTORC1 functions that involve autophagy regulation are resistant to inhibition by rapamycin (Thoreen and Sabatini, 2009). Also, the differential effects of Torin1 and rapamycin on TFEB regulation and lysosomal functions are consistent with previous studies (Thoreen and Sabatini, 2009; Zhou et al., 2013). For instance, it has been shown that Torin1 is a potent activator of TFEB nuclear translocation, whereas rapamycin has little or no effect on TFEB subcellular localization (Zhou et al., 2013; Settembre et al., 2012; Li et al., 2018). It has also been shown that the TFEB Ser142 residue is a rapamycin-resistant mTORC1 phosphorylation site, and that Torin1, but not rapamycin, is able to block nutrient-induced phosphorylation of TFEB on Ser142 (Settembre et al., 2012). Moreover, it was found that rapamycin is unable to increase TFEB transcriptional activity, and that suppression of mTOR activity by Torin1, but not rapamycin, leads to activation of lysosomal function (Zhou et al., 2013). Thus, our results further support that TFEB is a rapamycin-resistant substrate of mTORC1. Our previous finding that rapamycin treatment resulted in decreased survival of neuronopathic GD neurons can be explained by the inability of rapamycin to improve lysosomal functions in those cells (Awad et al., 2015). Although mTOR inhibition by rapamycin induced autophagosome formation in GD iPSC neurons, it was not associated with increased autophagosome-lysosome fusion or improvement in autophagic clearance (Awad et al., 2015). Here, we found that Torin1 did increase autophagosome-lysosome association and improved autophagic clearance in neuronopathic GD neurons and that these effects were likely mediated through enhancement of TFEB activity. It would be interesting to investigate the effect of Torin1 on the survival of GD neurons in future studies.

Although our study focuses on the effect of *GBA1* mutations on TFEB regulation by mTOR, it does not explore other upstream regulatory signals, which may also be affected by *GBA1*. One potential pathway is the unfolded protein response and endoplasmic reticulum stress, which is shown to activate TFEB through PERK (PKR-like endoplasmic reticulum kinase) and calcineurin, but is independent of mTOR (Martina et al., 2016).

In conclusion, our study provides a novel mechanism contributing to autophagy-lysosomal pathway dysfunction in neuronopathic GD and identifies the mTOR complex as a potential therapeutic target in *GBA1*-associated neurodegeneration.

## MATERIALS AND METHODS

### Generation of neuronopathic GD iPSC NPCs and neurons

All the control and GD iPSC lines used in this study have been previously described (Sgambato et al., 2015; Panicker et al., 2012; Awad et al., 2017). The DF4-7T.A (7TA) iPSC line and the hESC line H9/WA09 were purchased from the WiCell Repository. The neuronopathic GD iPSC lines were derived from two neuronopathic type 2 GD patients harboring the bi-allelic mutations W184R/D409H (GD2a) and L444P/Rec*Nci*I (GD2b) and from one neuronopathic type 3 GD patient with L444P/L444P mutations (GD3). Experiments were performed using the three neuronopathic GD iPSC lines and data from GD2 and GD3 were combined where indicated in the figure legends. For each neuronopathic GD iPSC line, two clones were generated and assayed. NPCs were generated from embryoid bodies after neuronal induction and rosette formation as previously described (Awad et al., 2015). NPCs were maintained in culture media consisting of Neurobasal medium (Life Technologies), containing 1× (vol/vol) MEM non-essential amino acids (Life Technologies), 1× (vol/vol) GlutaMAX-I CTS (Life Technologies), 1× (vol/vol) B27 supplement (Life Technologies), 1× (vol/vol) penicillin/streptomycin and 20 ng/ml basic fibroblast growth factor (Stemgent), with media changed every other day. NPCs were differentiated to neurons according to our previously described protocol and maintained in neuronal differentiation medium for 3-4 weeks before being assayed (Awad et al., 2015). All control and GD iPSC NPCs used in the study had been recently tested for contamination.

### Immunofluorescence analysis

For immunofluorescence analysis, NPCs were plated on chamber slides (Lab-Tek) or differentiated into neurons on poly-L-ornithine/laminin-coated glass-bottom culture dishes (MatTek). Cells were fixed in 4% (vol/vol) paraformaldehyde for 15 min followed by blocking in phosphate-buffered saline (PBS) containing 8% fetal bovine serum (vol/vol) for 1 h. The primary antibodies were prepared in PBS solution with 2 mg/ml saponin and incubated for 2 h at room temperature or overnight at 4°C. The cells were then washed in PBS and incubated with the corresponding fluorochrome-conjugated secondary antibodies for 1 h. Vectashield mounting medium plus DAPI (Vector Laboratories) was applied to label the nuclei. The following primary antibodies were used: anti-mTOR (Cell Signaling Technology, 2972) 1:100; anti-phospho-mTOR-Ser2448 (Cell Signaling Technology, 5536) 1:100; anti-S6 Ribosomal Protein (Cell Signaling Technology, 2217) 1: 200; anti-phosphoS6-Ser235/236 (Cell Signaling Technology, 2211) 1:100; anti-phospho-4EBP1-Thr37/46 (Cell Signaling Technology, 2855) 1:100; anti-LAMP1 (Developmental Studies Hybridoma Bank, H4A3) 1:200; anti-p62 (BD Biosciences, 610832) 1:100; anti-Tuj1 (Neuromics, MO15013) 1:200; anti-TFEB (MyBioSource, MBS855552) 1:50. The secondary antibodies used were Alexa fluor 488- or 594-conjugated mouse or rabbit (Life Technologies), both at a 1:200 or 1:400 dilution.

### Chemical reagents and cell treatment

For mTOR pathway analysis, NPCs and neurons were treated with 200 nM rapamycin (Sigma-Aldrich) or 100 nM Torin1 (Sigma-Aldrich) for 2-6 h. NPCs were also treated with 10 nM insulin (Sigma-Aldrich) for 20-40 min where indicated. For lysosomal biogenesis and autophagy studies, cultured neurons were treated with the same dose of rapamycin or Torin1 as above for 18-24 h before analysis. For substrate reduction treatment, we treated NPCs with GZ-161. This compound was synthesized according to WO 2012/129084 at The Moulder Center for Drug Discovery Research, Temple University School of Pharmacy, Philadelphia, PA, USA. NPCs were incubated with 5 µM GZ-161 for 72 h before being assayed. We also used the proteasome inhibitor, Clasto-lactacystin β-lactone (Cayman Chemical), at a concentration of 0.5 μg/ml for 18 h where indicated.

### Lentiviral infection

The TFEB-GFP and GFP-LC3 lentiviral constructs used in this study have been previously described (Awad et al., 2015). For lentiviral infection, control and GD neurons were incubated with the lentiviral particles in media containing 6 μg/ml Polybrene. After 48 h the culture media was replaced with fresh media and GFP expression was analyzed. Three days after infection, neurons expressing TFEB-GFP or GFP-LC3 fusion proteins were treated with 200 nM rapamycin or 100 nM Torin1 for 18-24 h as indicated. Neuronal cultures were then fixed in 4% paraformaldehyde and immune-labeled with anti-LAMP1 or anti-p62 antibodies, followed by fluorescence image acquisition and analysis.

### Fluorescence images acquisition and quantitation

Fluorescence images were captured using an inverted Nikon Eclipse TE-2000 microscope, a Nikon Ti-E inverted microscope with Nikon Imaging Systems (NIS)-Elements, or a Revolve microscope with Olympus optics and 8MP color camera (Echo Laboratories). Images were analyzed using ImageJ software (National Institutes of Health), after background subtraction and threshold detection. For LAMP1 fluorescence signal quantitation, *z*-stack images were acquired using a Nikon Ti-E inverted microscope at 60× (CFI Plan APO VC 60× NA 1.4 Oil) and focused using the extended depth of focus (EDF) module of the Nikon Elements software. All images within each experiment were acquired at the same microscope settings. A minimum of 50 neurons were analyzed per cell line in at least four high power fields (HPF) per experiment. The percentage of neurons with TFEB-GFP-positive nuclei was calculated as the number of cells with TFEB-GFP fluorescence signal that colocalized with nuclear DAPI, divided by the number of GFP-positive cells in the same vision field. The ratio of nuclear/cytoplasmic TFEB-GFP signal was quantified using Nikon Elements software. The nuclei were defined based on DAPI staining using spot detection; TFEB-GFP intensity was quantified in areas overlapping versus non-overlapping with DAPI and expressed as a ratio. For GFP-LC3 puncta analysis, images were acquired using a Nikon Ti-E inverted microscope (CFI Plan APO VC 20× NA 0.75 WD 1 mm) or a Revolve microscope (UCPLFLN 40× or 20×), and analyzed using ImageJ software after background subtraction and threshold detection. The number and fluorescence intensity of GFP-LC3 puncta were analyzed in at least 100 neurons per group in 3-4 fields in each experiment and normalized to the number of GFP-expressing cells in the same field of vision. GFP-LC3 puncta-LAMP1 fluorescence signal colocalization was determined by measuring the percentage of overlap in the area of integrated intensity for the two markers, which corresponds to the colocalization coefficient (Manders et al., 1993).

### Western blot analysis

iPSC-derived NPCs and neurons were lysed in RIPA buffer supplemented with protease inhibitor (Roche) and phosphatase inhibitors (Pierce), or directly lysed in Laemmli buffer (Bio-Rad), followed by sonication. Protein extracts were denatured in loading buffer at 95°C for 5 min before loading onto 4-20% polyacrylamide gels (Bio-Rad) and analyzed by electrophoresis. The proteins were then transferred to PVDF or nitrocellulose membranes and incubated overnight with the primary antibodies at 4°C, followed by incubation with the corresponding HRP-conjugated secondary antibody for 1 h. Membranes were developed using SuperSignal West Pico PLUS Chemiluminescent Substrate (Thermo Fisher Scientific) and imaged using a Chemidoc imager and Imagelab software (Bio-Rad). Primary antibodies used were: anti-mTOR, anti-phospho-mTOR-Ser2448, anti-S6 Ribosomal Protein, anti-phospho-S6-Ser235/236, anti-phospho-4EBP1-Thr37/46, anti-LAMP1, anti-p62 (all at 1:1000), anti-NBR1 (Cell Signaling Technology, 9891, 1:1000), anti-TFEB (MyBioSource, MBS855552, 1:1000), anti-phosphoTFEB-Ser142 (Millipore, ABE1971, 1:500), anti-ubiquitin (Cell Signaling Technology, 3933, 1:1000), and anti-β-actin (Sigma-Aldrich, 1:4000).

### Immunoprecipitation (IP)

NPCs were lysed in IP lysis buffer, followed by pre-clearing the lysate for 1 h with Protein A/G/Agarose Beads (Thermo Fisher Scientific, 20421). The cell lysates were then incubated overnight with the primary antibody at 4°C. The following day, Protein A/G Agarose Beads were added and incubated with rotation for 2 h. Beads were then washed and protein was eluted for western blotting. The primary antibody used was anti-phospho-TFEB-Ser142 at 1:200 dilution.

### Real-time PCR

For gene expression analysis, neurons were cultured in 12-well plates and treated with rapamycin or Torin1 as described above. mRNA was extracted using the RNAeasy kit (Qiagen). cDNA was synthesized using iScript cDNA synthesis kit (Bio-Rad). Gene expression was determined by quantitative PCR (7900 HT, Applied Biosystems) in duplicate wells using SYBR Green PCR Master Mix (Thermo Fisher Scientific). Relative gene expression was normalized to the corresponding value of *GAPDH* expression, and the fold changes relative to the control values within the same experiment were determined. The sequences of the primers are listed in Table S1.

### TFEB-GFP H4 neuroglioma cells

The neuroglioma cell line H4 (ATCC), was infected with TFEB-GFP lentiviral vector (previously described) (Awad et al., 2015), and selected for stable TFEB-GFP expression using 1 µg/ml Puromycin. Cells were maintained in DMEM supplemented with 10% fetal bovine serum and 1% penicillin/streptomycin. Cells were treated with 1 mM conduritol B-epoxide (CBE) (Sigma-Aldrich) for 48 h alone or with the proteasome inhibitor Clasto-lactacystin β-lactone as indicated. Cells were either lysed for western blot analysis or imaged for GFP fluorescence expression.

### Statistical analysis

Data are mean±s.e.m and analyzed using one-way ANOVA followed by Tukey's or Sidak's post-test to determine statistical differences between multiple groups. Two-tailed unpaired Student's *t*-tests were used for comparison between two groups when appropriate. *P*-values <0.05 were considered statistically significant. The confidence level for significance was 95%. Data were analyzed using Prism software version 7.0a (GraphPad Software).

## Supplementary Material

Supplementary information
