# Supplementary material for: mTOR hyperactivity mediates lysosomal dysfunction in Gaucher's disease iPSC-neuronal cells
Source: Dis Model Mech. 2019 Oct 16;12(10):dmm038596. doi: 10.1242/dmm.038596 (PMC6826018; doi:10.1242/dmm.038596)
Supplement: Supplementary information [file dmm-12-038596-s1.pdf]

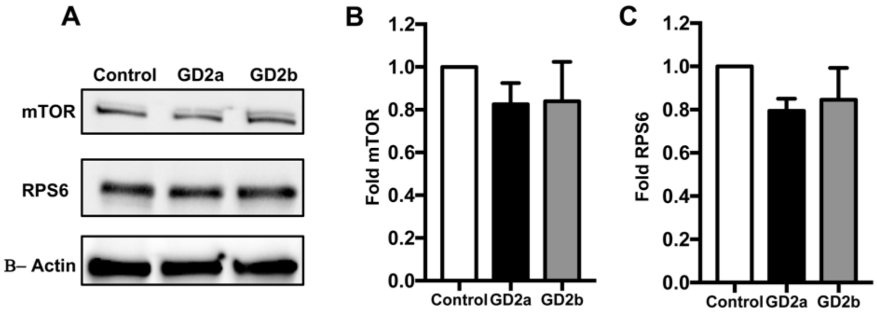

Figure S1. Levels of mTOR and RPS6 in GD NPCs. A) Representative western blot showing mTOR and RPS6 protein levels in control and GD2 NPCs. B) Bar graph represents fold mTOR in GD2 NPCs (GD2a and GD2b combined) relative to control. Data are mean ±s.e.m, *n*= 3-4 per group. $p>0.05$ between control and GD2a and GD2b as assessed by Student's *t*-test.

C) Bar graph represents fold RPS6 in GD2 NPCs (GD2a and GD2b combined) relative to control. Data are mean ±s.e.m, *n*= 3-4 per group. $p>0.05$ between control and GD2a and GD2b as assessed by Student's *t*-test.

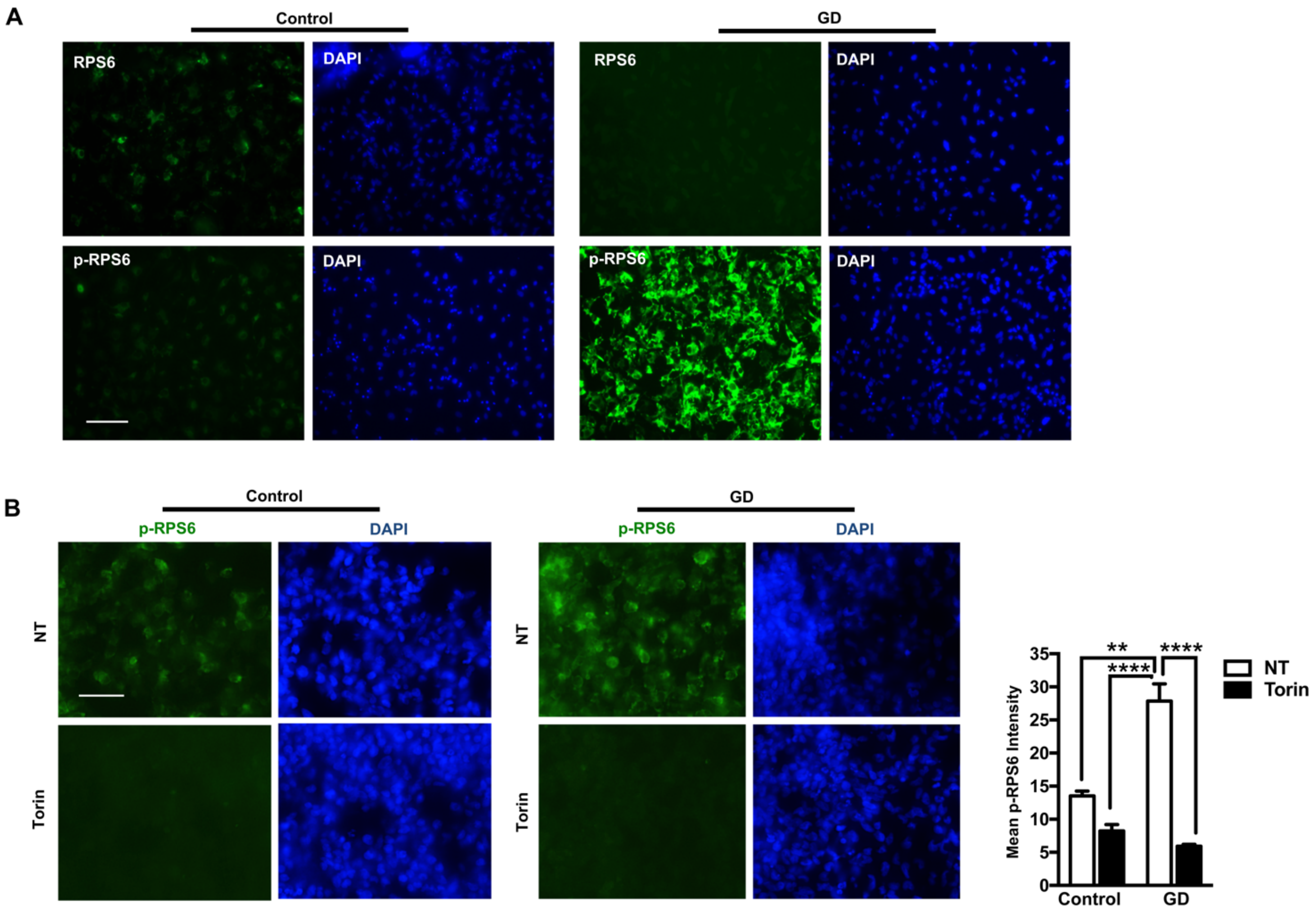

Figure S2. Increased p-RPS6 protein levels in GD NPCs. A) Representative immunofluorescence images of control and GD2b NPCs labeled with antibodies to RPS6 (top) or p-RPS6 (bottom) and DAPI. Magnification 20x; scale bar, 100 µm. B) Representative immunofluorescence images of control and GD2a NPCs labeled with an antibody to p-RPS6 and DAPI. Cells were either untreated (NT) or treated with 100 nM Torin1 for 6 hours. Magnification 20x; scale bar, 100 µm. Bar graph to the right represents mean p-RPS6 fluorescence signal intensity in control and GD NPCs (GD2a and GD2b combined) with and without Torin1. Data were collected from >100 cells per group, assayed in 3-5 different fields in a representative experiment. Error bars = s.e.m., $**p<0.005$ and $****p<0.00005$ between the indicated groups as assessed by One-way ANOVA.

Disease Models & Mechanisms • Supplementary information

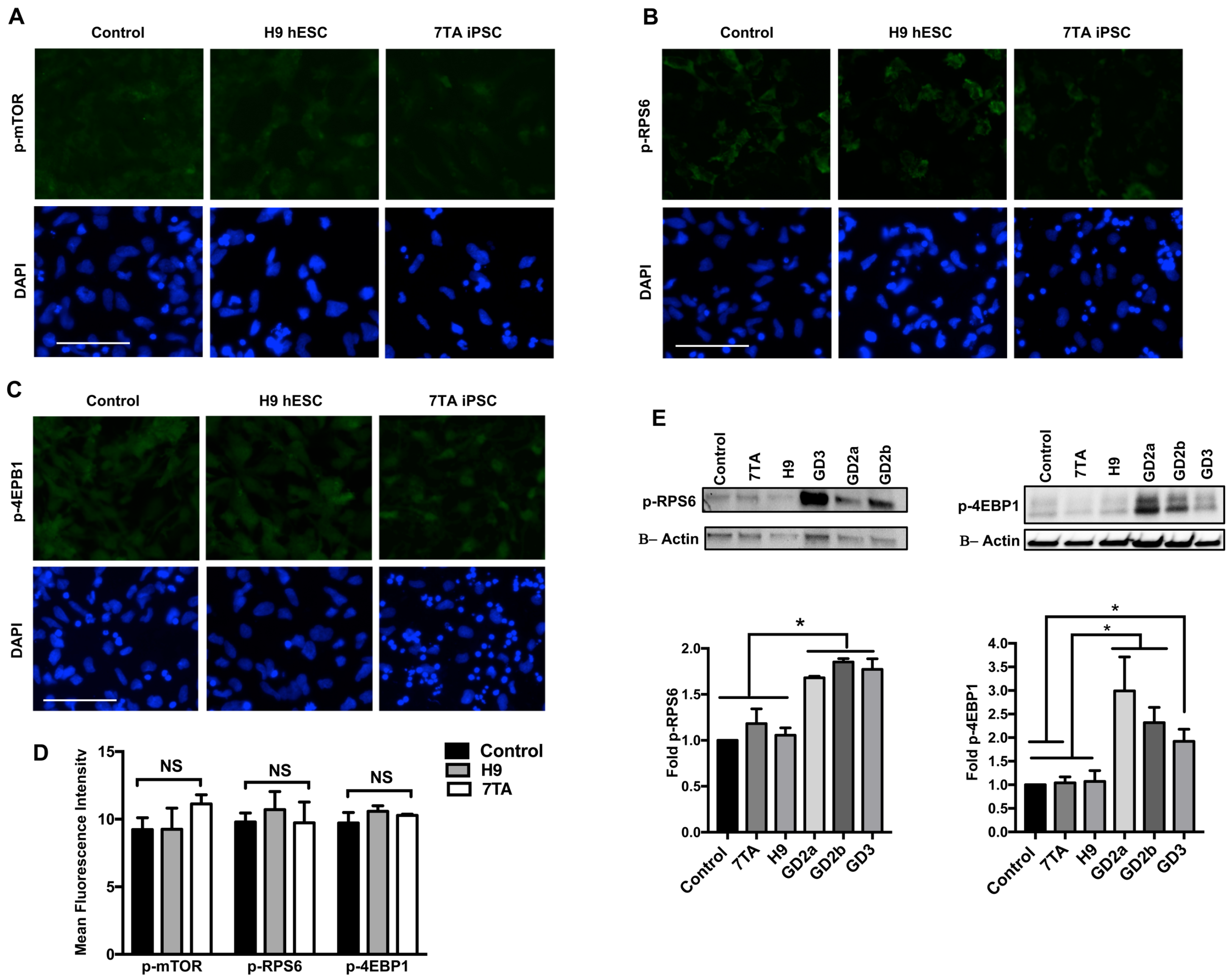

Figure S3. Increased mTORC1 activity in GD NPCs. A) Representative immunofluorescence images of NPCs generated from control, H9 hESC and 7TA iPSC, labeled with an antibody to p-mTOR (top) and DAPI (bottom). Magnification 20x; scale bar, 100 μm. B) Representative immunofluorescence images of NPCs from control, H9 hESC and 7TA iPSC, labeled with an antibody to p-RPS6 (top) and DAPI (bottom). Magnification 20x; scale bar, 100 μm. C) Representative immunofluorescence images of NPCs from control, H9 hESC and 7TA iPSC labeled with an antibody to p-4EPB1 (top) and DAPI (bottom). Magnification 20x; scale bar, 100 μm. D) Quantitation of p-mTOR, p-RPS6 and p-4EBP1 fluorescence signal intensity in control, H9 hESC and 7TA iPSC. Data were collected from >200 cells per group, assayed in 2-4 different fields in a representative experiment. No statistically significant difference (NS) between the indicated groups as assessed by One-way ANOVA, $p>0.05$. E) Representative western blot showing p-RPS6 and p-4EPB1 levels in NPCs from control, 7TA iPSC, H9 hESC and GD iPSCs (GD2a, GD2b and GD3). Bar graphs below represent fold p-RPS6 and fold p-4EPB1 relative to control. Data are mean ±s.e.m, n= 3-5 per group. *$p<0.05$ between the indicated groups as assessed by Student's t-test.

Disease Models & Mechanisms • Supplementary information

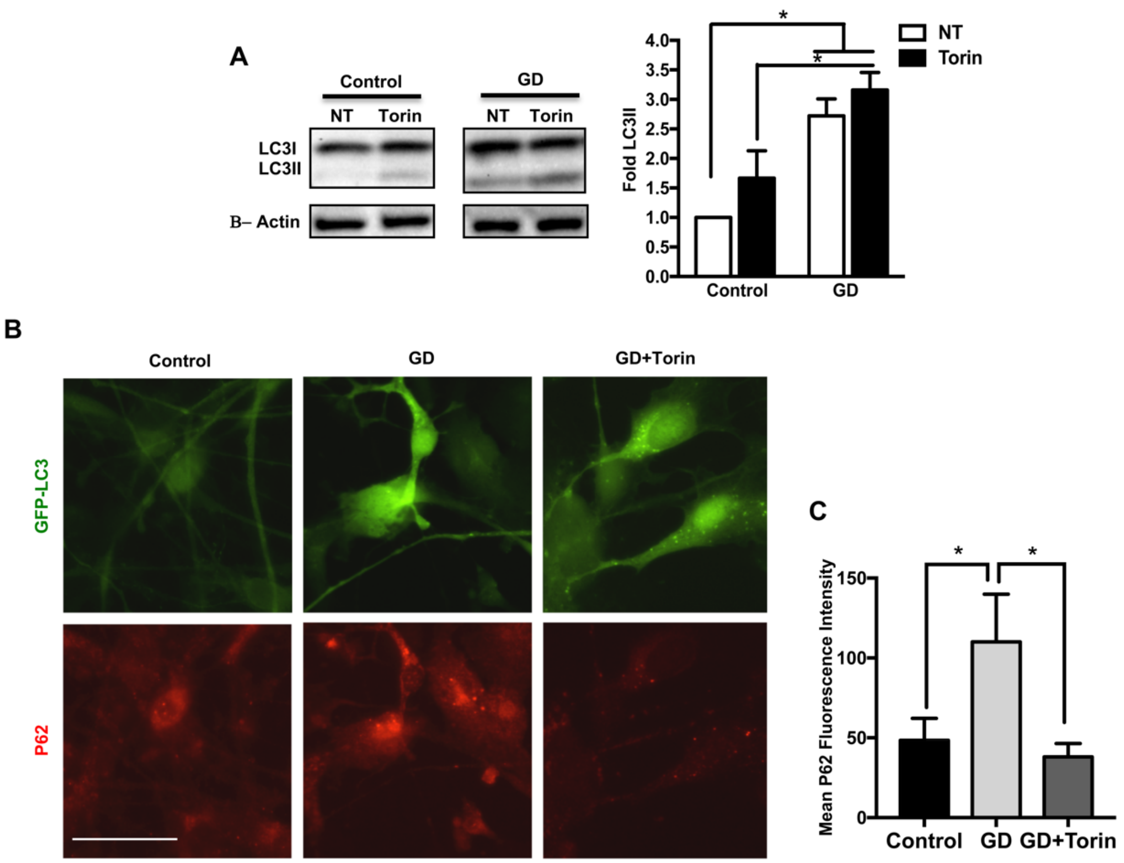

Figure S4. Effect of Torin1 treatment on LC3 and p62 protein levels in GD neuronal cells. A) Representative western blot showing LC3 levels in control and GD2b NPCs with or without 100 nM Torin1 treatments for 6 hours. Also shown is β–actin loading control. Bar graph represents fold LC3II in GD2 NPCs (GD2a and GD2b combined) relative to untreated (NT) control. Data are mean ±s.e.m., *n*= 3 per group. **p*<0.05 between the indicated groups as assessed by One-way ANOVA. B) Representative fluorescence images for control and GD2a neurons, expressing GFP-LC3 fusion protein and labeled with anti-p62 antibody. GD2a neurons were either untreated or treated with 100 nM Torin1 for 18 hours. Magnification 20x; scale bar, 50 µm. C) Fluorescence quantitation of p62 fluorescence signal intensity in control and GD2a neurons. Compiled data from >50 cell per group assayed in at least 4 different fields in a representative experiment. Bar graph represents p62 mean fluorescence intensity. Error bars = s.e.m., **p*<0.05 between the indicated groups as assessed by Student's *t*-test.

Disease Models & Mechanisms • Supplementary information

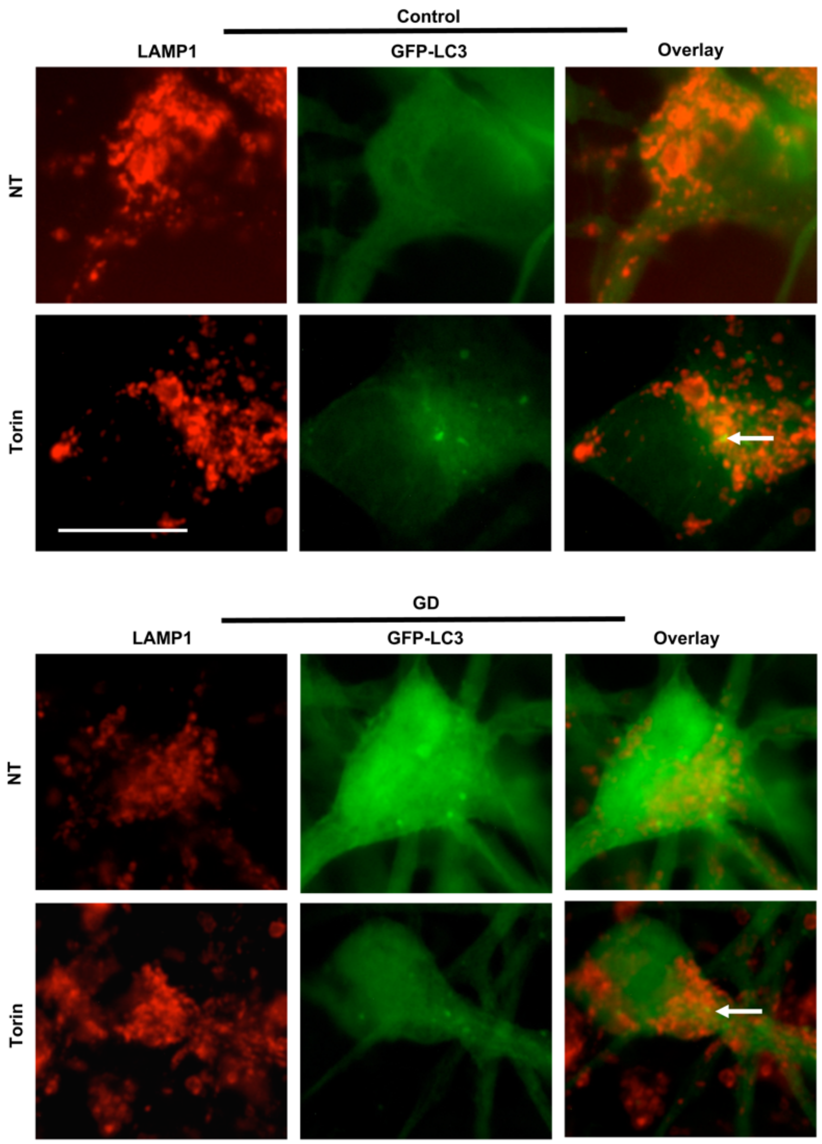

Figure S5. Torin1 increases autophagosome-lysosomal association in GD neurons.

Representative fluorescence images for control and GD2b neurons, expressing GFP-LC3 fusion protein and labeled with anti-LAMP1 antibody. Neurons were either untreated or treated with 100 nM Torin1 for 18 hours. The last panel in each group shows overlay of GFP-LC3 and LAMP1 fluorescence signal. Arrows point to co-localization of the GFP-LC3 puncti (green) and LAMP1-labeled lysosomes (red) in neurons treated with Torin1. Fluorescence images for LAMP1 in neurons treated with Torin1 were acquired using lower exposure times to avoid signal over-saturation. Magnification 40x; scale bars, 20 μm.

Disease Models & Mechanisms • Supplementary information

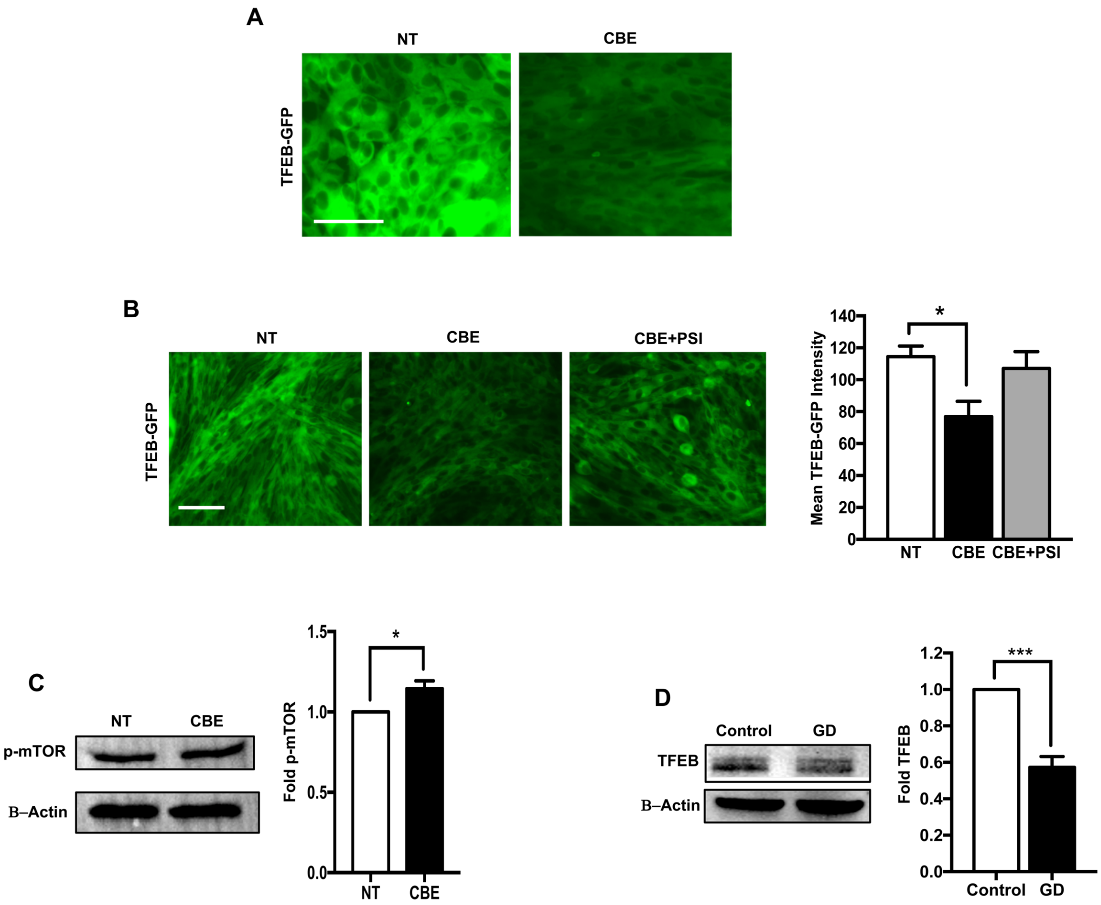

Figure S6. Decreased GCase activity affects TFEB stability. A) Representative fluorescence images for H4 cells expressing TFEB-GFP fusion protein that were either untreated (NT) or treated with 1mM conduritol B-epoxide (CBE) for 48 hours. Magnification 10x; scale bar, 100 µm. B) Representative fluorescence images for H4 cells expressing TFEB-GFP fusion protein that were either untreated (NT), treated with CBE, or co-treated with CBE and with the proteasome inhibitor, Clasto-lactacystin β-lactone for 48 hours. Magnification 10x; scale bar, 100 µm. Bar graph represents TFEB-GFP mean fluorescence intensity in H4 cells untreated (NT), treated with CBE, or co-treated with CBE and PSI for 48 hours in a representative experiment ±s.e.m., *p<0.05 as assessed by Student's *t*-test. C) Representative western blot showing p-mTOR levels in TFEB-GFP expressing H4 cells that were either untreated (NT) or treated with CBE for 48 hours. Also shown is β–actin loading control. Bar graph represents fold p-mTOR in cells treated with CBE relative to untreated cells (NT). Data are mean ±s.e.m., n= 3 per group. *p<0.05 as assessed by Student's *t*-test. D) Representative

western blot showing TFEB levels in control and GD3 NPCs. Also shown is β–actin loading control. Bar graph represents fold TFEB in GD NPCs (GD2a, GD2b and GD3 combined) relative to control. Data are mean ±s.e.m., n= 4 per group. ***p<0.0005 as assessed by Student's *t*-test.

Table S1: Sequences of qRT-PCR primers used in the study.

GBA -F	TGGGTACCCGGATGATGTTA
GBA-R	AGATGCTGCTGCTCTCAACA
HEXA-F	CAACCAACACATTCTTCTCCA
HEXA-R	CGCTATCGTGACCTGCTTTT
LAMP1-F	ACGTTACAGCGTCCAGCTCAT
LAMP1-R	TCTTTGGAGCTCGCATTGG
CTSD-F	AACTGCTGGACATCGCTTGCT
CTSD-R	CATTCTTCACGTAGGTGCTGGA
CTSB-F	AGTGGAGAATGGCACACCCTA
CTSB-R	AAGAAGCCATTGTCACCCCA
GNS-F	CCCATTTTGAGAGGTGCCAGT
GNS-R	TGACGTTACGGCCTTCTCCTT
GAPDH-F	CAAGATCATCAGCAATGCCT
GAPDH-R	CTTCCACGATACCAAAGTTGTC
